# Recurrent evolution and selection shape structural diversity at the amylase locus

**DOI:** 10.1038/s41586-024-07911-1

**Published:** 2024-09-04

**Authors:** Davide Bolognini, Alma Halgren, Runyang Nicolas Lou, Alessandro Raveane, Joana L. Rocha, Andrea Guarracino, Nicole Soranzo, Chen-Shan Chin, Erik Garrison, Peter H. Sudmant

**Affiliations:** 1 Human Technopole, Milan, Italy; 2 Department of Integrative Biology, University of California Berkeley, Berkeley, CA USA; 3 Department of Genetics, Genomics, and Informatics, University of Tennessee Health Science Center, Memphis, TN USA; 4 Wellcome Sanger Institute, Hinxton, UK; 5 National Institute for Health Research Blood and Transplant Research Unit in Donor Health and Genomics, University of Cambridge, Cambridge, UK; 6 Department of Haematology, Cambridge Biomedical Campus, Cambridge, UK; 7 British Heart Foundation Centre of Research Excellence, University of Cambridge, Cambridge, UK; 8 Foundation for Biological Data Science, Belmont, CA USA; 9 Center for Computational Biology, University of California Berkeley, Berkeley, CA USA

**Keywords:** Evolutionary biology, Evolutionary genetics, Genome evolution, Structural variation, Molecular evolution

## Abstract

The adoption of agriculture triggered a rapid shift towards starch-rich diets in human populations^1^. Amylase genes facilitate starch digestion, and increased amylase copy number has been observed in some modern human populations with high-starch intake^2^, although evidence of recent selection is lacking^3,4^. Here, using 94 long-read haplotype-resolved assemblies and short-read data from approximately 5,600 contemporary and ancient humans, we resolve the diversity and evolutionary history of structural variation at the amylase locus. We find that amylase genes have higher copy numbers in agricultural populations than in fishing, hunting and pastoral populations. We identify 28 distinct amylase structural architectures and demonstrate that nearly identical structures have arisen recurrently on different haplotype backgrounds throughout recent human history. *AMY1* and *AMY2A* genes each underwent multiple duplication/deletion events with mutation rates up to more than 10,000-fold the single-nucleotide polymorphism mutation rate, whereas *AMY2B* gene duplications share a single origin. Using a pangenome-based approach, we infer structural haplotypes across thousands of humans identifying extensively duplicated haplotypes at higher frequency in modern agricultural populations. Leveraging 533 ancient human genomes, we find that duplication-containing haplotypes (with more gene copies than the ancestral haplotype) have rapidly increased in frequency over the past 12,000 years in West Eurasians, suggestive of positive selection. Together, our study highlights the potential effects of the agricultural revolution on human genomes and the importance of structural variation in human adaptation.

## Abstract (web summary)

The impact of structural variation on the evolution of the amylase genes is explored using human pangenome resources and ancient DNA data.

## Main

Dietary changes have had a major role in human adaptation and evolution, impacting phenotypes such as lactase persistence^5,6^ and polyunsaturated fatty acid metabolism^7–9^. One of the most substantial recent changes to the human diet is the shift from hunter-gatherer societies to agricultural-based subsistence. The earliest instance of crop domestication can be traced to the Fertile Crescent of southwestern Asia approximately 12 thousand years before present (kyr bp), laying the foundation for the Neolithic revolution^1^. Agriculture subsequently spread rapidly westward into Europe by way of Anatolia by approximately 8.5 kyr bp and eastward into the Indian subcontinent. However, the transition to agriculture-based subsistence has happened independently several other times throughout human history, and today, the overwhelming majority of carbohydrates consumed by humans are derived from agriculture.

Plant-based diets are rich in starches, which are broken down into simple sugars by α-amylase enzymes in mammals. Human genomes contain three different amylase genes located proximally to one another at a single locus: *AMY1*, which is expressed exclusively in salivary glands, and *AMY2A* and *AMY2B*, which are expressed exclusively in the pancreas. However, it has long been appreciated that the amylase locus exhibits extensive structural variation in humans^10,11^, with all three genes exhibiting copy number variation. Indeed, the haplotype represented in the human reference genome GRCh38 contains three tandemly duplicated *AMY1* copies (see the Methods for details on amylase gene naming conventions). Other great apes do not exhibit copy number variation and have just a single copy each of the *AMY1*, *AMY2A* and *AMY2B* genes^12^. These three amylase genes are the result of duplication events, occurring first in the common ancestor of Old World monkeys and apes, and again in the common ancestor of great apes^13^. This ancestral single-copy state has also been reported in Neanderthals and Denisovans^3^. *AMY1* copy number correlates with salivary amylase protein levels in humans, and an analysis of seven human populations found increased *AMY1* copy number in groups with high-starch diets^2^. Although it has been proposed that this gene expansion may have been an adaptive response to the transition from hunter-gatherer to agricultural societies, evidence of recent selection at this locus has been lacking^3,4^. Moreover, subsequent analyses identifying a putative association of *AMY1* copy number and body mass index^14^ failed to replicate^15^, highlighting the challenges associated with studying structurally variable loci, which are often poorly tagged by nearby single-nucleotide polymorphisms (SNPs)^16^. Another major challenge in characterizing selective signatures at structurally complex loci is the difficulty of phasing copy numbers onto haplotypes. Furthermore, although the human reference genome contains a single fully resolved amylase haplotype, the sequence, structure and diversity of haplotypes on which different copy numbers have emerged are unknown.

## Amylase copy number diversity worldwide

**Fig. 1:**
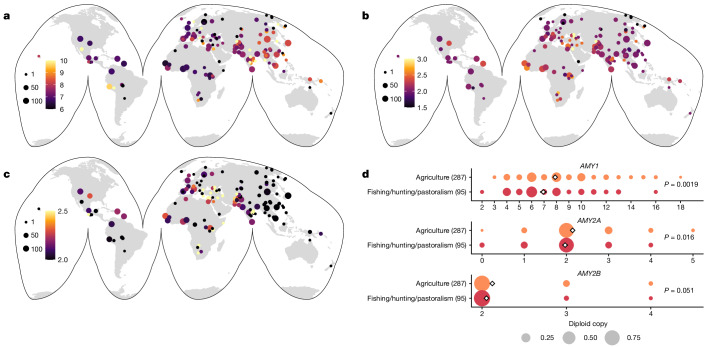
Worldwide amylase copy number diversity. **a**–**c**, World maps indicating average *AMY1* (**a**), *AMY2A* (**b**) and *AMY2B* (**c**) copy number in 147 different human populations. The point size indicates population sample sizes (ranging from 1 to 134), and the colour indicates the mean copy number. Copy number distributions across individual populations and continental groups are displayed in Extended Data Fig. 1. **d**, Copy number distributions of *AMY1* (top), *AMY2A* (middle) and *AMY2B* (bottom) in 33 modern human populations with traditionally agricultural subsistence compared with fishing-based, hunting-based and pastoralism-based diets. Numbers in parentheses indicate sample size. Two-sided *P* values of a Student’s *t*-test are shown without adjustment for multiple comparisons.

**Extended Data Fig. 1:**
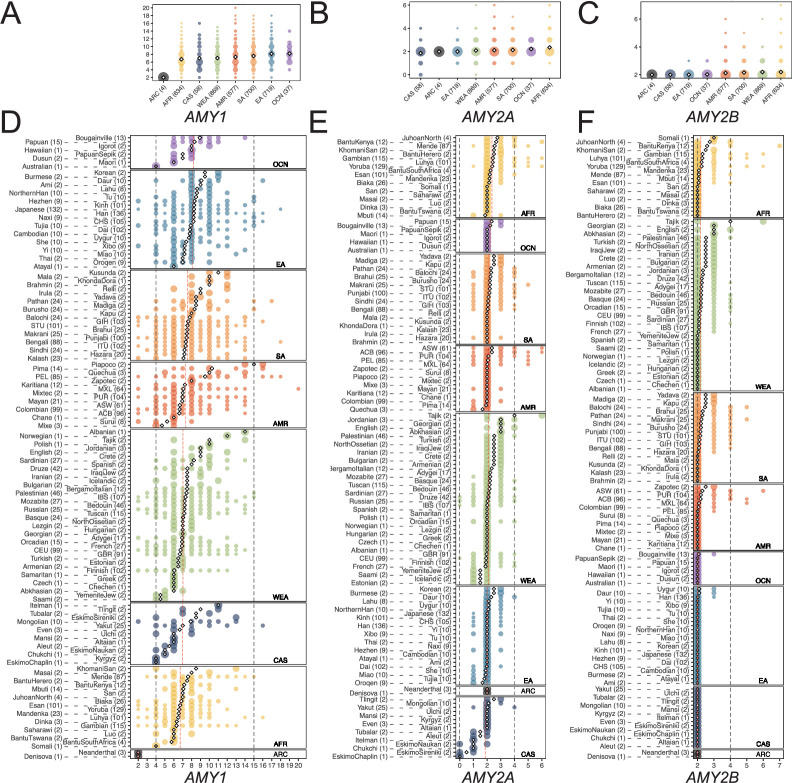
Worldwide amylase subpopulation copy number diversity. **A**-**C**) Copy number distributions of *AMY1, AMY2A*, and *AMY2B* in continental populations and **D-F**) in 147 modern human populations and four archaic hominids. The size of each point is proportional to the proportion of individuals in the population with that genotype. Diamonds indicate the subpopulation mean, red dashed lines indicate the continental population mean, grey dashed lines indicate minimum and maximum subpopulation means.

Although extensive copy number variation has been documented at the amylase locus in humans^3,14,15,17^, sampling of human diversity worldwide has been incomplete. To explore diversity at this locus, we compiled 4,292 diverse high-coverage modern genomes from several sources^18–20^ (see the Methods for information on all datasets used in this paper) and used read-depth-based approaches (see the Methods; Supplementary Fig. 1) to estimate diploid copy number in 147 different human populations (Fig. 1a–c, Extended Data Fig. 1 and Supplementary Table 1, subcontinental groupings as per Mallick et al.^20^). Diploid *AMY1* copy number estimates ranged from 2 to 20 and were highest in populations from Oceanic, East Asian and South Asian subcontinents. Nevertheless, individuals carrying high *AMY1* copy numbers were present in all continental subgroups. *AMY2A* (0–6 copies) showed the highest average copy number in African populations, with deletions more prevalent in non-African populations. *AMY2B* (2–7 copies) exhibited high-population stratification with duplications essentially absent from Central Asian/Siberian, East Asian and Oceanic populations. We also assessed three high-coverage Neanderthals and a single Denisovan individual, confirming all to have the ancestral copy number state (Extended Data Fig. 1). Thus, copy number variation across all three amylase genes is probably human specific.

**Extended Data Fig. 2:**
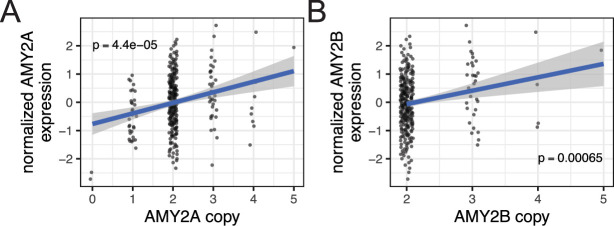
GTEx gene expression. Comparison between copy number and gene expression for **A**) *AMY2A* and **B**) *AMY2B* in the pancreas across 305 individuals. A linear model is fit to the data and 95% confidence intervals are indicated by shades. Two-sided p-values from the linear model are shown without adjustment for multiple testing.

Although *AMY1* copy number has been shown to exhibit a strong positive correlation with salivary protein levels^2^, the relationship between pancreatic amylase gene expression and copy number has not been assessed. Analysing GTEx^21^ data, we confirmed that *AMY2A* and *AMY2B* expression was confined to the pancreas. We then genotyped diploid copy numbers in 305 samples for which expression data were available alongside high-coverage genome sequencing. Both *AMY2A* (0–5 copies) and *AMY2B* (2–5 copies) copy numbers were significantly and positively correlated with gene expression levels (*P* = 4.4 × 10^−5^ and *P* = 6.5 × 10^−4^, respectively, linear model; Extended Data Fig. 2).

The strongest evidence of potential selection at the amylase locus comes from comparisons of seven modern-day populations with high-starch versus low-starch intake^2^. We identified 382 individuals from 33 different populations with traditionally agricultural-based, hunter-gatherer-based, fishing-based or pastoralism-based diets in our dataset (Supplementary Table 2). The copy number of all three amylase genes was higher in populations with agricultural subsistence than in those from fishing, hunting and pastoral groups, although it was only strongly significant for *AMY1* (Fig. 1d and Supplementary Fig. 2; *P* = 0.0019, *P* = 0.016 and *P* = 0.051 for *AMY1*, *AMY2A* and *AMY2B*, respectively, Student’s *t*-test). These results thus corroborate previous work and demonstrate that pancreatic amylase gene duplications are also more common in populations with starch-rich diets.

## Twenty-eight distinct structural haplotypes

**Fig. 2:**
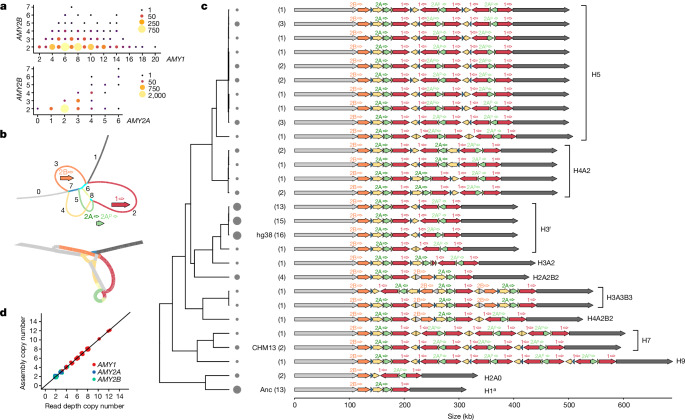
Pangenome-based identification of amylase structural haplotype diversity. **a**, The relationship between *AMY1*, *AMY2A* and *AMY2B* copy number. The size and colour indicate the number of individuals with a copy number genotype pair. **b**, Hierarchical MAP-graph (top) and variation graph (bottom) architectures. The colours and numbers in the MAP-graph correspond to principal bundles shown in panel **c**. Genes associated with bundles are indicated. **c**, Twenty-eight distinct amylase structural haplotypes identified in 94 haplotypes. The filled arrows indicate principal bundles representing homology relationships, whereas labelled open arrows indicate genes (1 indicates *AMY1*, 2A indicates *AMY2A* and 2B indicates *AMY2B*). The numbers in parentheses and the circle sizes indicate the number of haplotypes identified with a specific structure. Haplotypes are ordered by their relationship in the tree (left), which is generated from the Jaccard distance between haplotypes from the variation graph. Consensus structures, which refer to clusters of similar structures, are indicated (right). The names of the consensus structures are formatted ‘H*x*A*y*B*z*’, where *x* corresponds to the copy number of *AMY1*, *y* to the number of *AMY2A*, and *z* to the number of *AMY2B*. ‘A*y*’ and ‘B*z*’ are only included in the name when *y* or *z* does not equal to 1. **d**, The relationship between read-depth-based copy number and assembly-based copy numbers for amylase genes for 35 individuals (70 haplotypes) in which both haplotypes were assembled across the amylase region.

The amylase structural haplotype present in the human reference genome (GRCh38) spans approximately 200 kb and consists of several long, nearly identical segmental duplications. Although the approximate structures of several other haplotypes have been inferred through in situ hybridization and optical mapping, these lack sequence and structural resolution^2,10,11,15^. Nevertheless, the variegated relationship between different amylase gene copy numbers (Fig. 2a) indicates the existence of a wide range of structures.

To characterize the structural diversity of the amylase locus, we first constructed a minimizer-anchored pangenome graph (MAP-graph)^22^ from 94 amylase haplotypes derived from 52 long-read, haplotype-resolved diploid genome assemblies recently sequenced by the Human Pangenome Reference Consortium (HPRC)^23^ alongside GRCh38 and T2T-CHM13 reference^24^ (see Methods; Fig. 2b). The MAP-graph captures large-scale sequence structures with vertices representing sets of orthologous or paralogous sequences; thus, input haplotypes can be represented as paths through the graph. We next performed a principal bundle decomposition of the graph, which identifies stretches of sequence that are repeatedly traversed by individual haplotypes (the coloured loops in Fig. 2b). These principal bundles represent the individual repeat units of the locus. We identified nine principal bundles in the amylase graph corresponding to: the unique sequences on either side of the structurally complex region containing amylase gene duplications (bundles 0 and 1), the repeat units spanning each of the three amylase genes and the *AMY2Ap* pseudogene (bundles 2, 3 and 5), as well as several other short repeat units (Fig. 2c). For 35 individuals in which both haplotypes were incorporated into the graph, short-read-based diploid genotypes were identical to the sum of the haplotype copy numbers, highlighting the concordance of both short-read genotypes and long-read haplotype assemblies (see Methods; Fig. 2d).

Together, we identified 28 unique structural haplotypes at the amylase locus (Fig. 2c and Supplementary Table 3), of which only 2 had been previously fully sequenced and characterized (the chimpanzee and human reference genome haplotypes). The structurally variable region (SVR) of the locus spans across all of the amylase genes and ranges in size from approximately 95 kb to approximately 471 kb, in all cases beginning with a copy of *AMY2B* and ending with a copy of *AMY1*. To better understand the relationships between these structural haplotypes, we constructed a pangenome variation graph using the PanGenome Graph Builder (PGGB)^25^ (Fig. 2b). In contrast to the MAP-graph, this graph enables base-level comparisons between haplotypes. Using this graph, we computed a distance matrix between all structural haplotypes and built a neighbour-joining tree from these relationships (see Methods; Fig. 2c). This tree highlights 11 different clusters of structures, or ‘consensus structures’, each defined by a unique copy number combination of amylase genes (Fig. 2c, right, the names of the consensus structures correspond to the copy number of *AMY1*, *AMY2A* or *AMY2B* genes; see the figure legend for details). Distinct structural haplotypes with the same consensus structure differed largely in the orientation of repeats, or only slightly in their composition. Several of these consensus structures correspond to approximate architectures that have been previously hypothesized^15^; however, three novel consensus structures are described here (H9, H3A2 and H3A3B3). Among these consensus structures, *AMY1* ranged from 1 to 9 copies with copy 6 and copy 8 states unobserved, *AMY2A* ranged from 0 to 3 copies, *AMY2Ap* ranged from 0 to 4 copies, and *AMY2B* ranged from 1 to 3 copies. We also assessed these haplotypes for mutations that might significantly disrupt the function of any of the amylase genes. We identified a single-base substitution that introduced a premature stop codon in *AMY1* shared between two haplotypes with high *AMY1* copy number, as well as several missense mutations in all three amylase genes of varying predicted impact (Supplementary Table 4). These mutations were generally found at low frequencies. Because of the low frequency (approximately 2%) and single origin of the loss-of-function mutation, we do not explicitly account for it in downstream analyses. Together, these results reveal the wide ranging and nested-nature of diversity at the amylase locus: different haplotypes can have vastly different copy numbers of each of the three genes, and haplotypes with identical gene copy numbers exist in a wide array of forms.

## Evolution of structural haplotypes

**Fig. 3:**
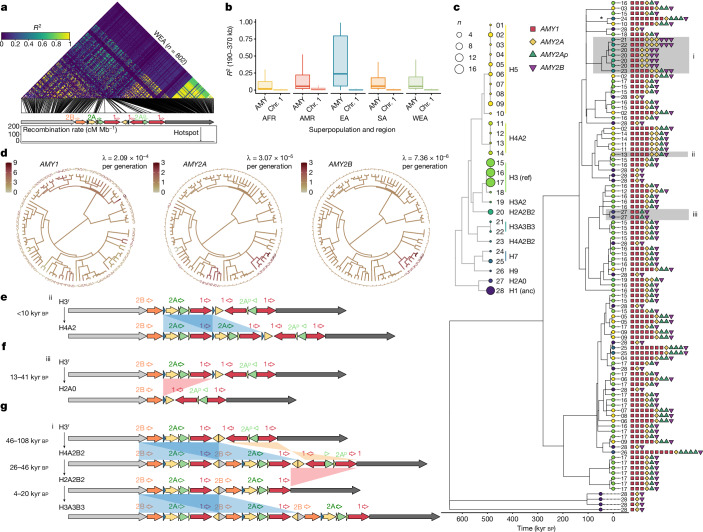
Evolutionary history of amylase structural haplotypes. **a**, Heatmap of linkage disequilibrium for SNPs across an approximately 406-kb region spanning unique sequences on either side of the structurally variable region of amylase for 802 West Eurasians (WEA; see Extended Data Fig. 3a for all populations). Schematics of the GRCh38 structure and the recombination rate are also shown (bottom). Note that regions outside the annotated recombination hotspot have recombination rates lower than 0.2 cM Mb^−1^. **b**, Boxplots comparing linkage disequilibrium between pairs of SNPs on either side of the SVR (that is, 190–370 kb apart) to identically spaced SNPs across chromosome 1 for major human populations with more than 100 samples. The centre line of the boxplot indicates the median, box limits indicate the first and third quartiles, and the whiskers indicate the smallest/largest observation within box limits ±1.5 times the interquartile range. AFR, Africa; AMR, Americas; CAS, Central Asia Siberia; EA, East Asia; OCN, Oceania; SA, South Asia. **c**, A time-calibrated coalescent tree from the distal non-duplicated region flanking the SVR (leftmost grey arrow in panel **a**) across 94 assembled haplotypes (the tree from the proximal region in Extended Data Fig. 4). The number next to each tip corresponds to the structural haplotype that the sequence is physically linked to, and the colour of the circle at each tip corresponds to its consensus haplotype structure (see the inset structural tree). The copy numbers of each amylase gene and pseudogene are also shown next to the tips of the tree. The asterisk indicates the single, recent origin of the premature stop codon in *AMY1*. **d**, Ancestral-state reconstruction and mutation rate estimates for amylase gene copy number (archaic outgroups are excluded). The branch colour corresponds to the copy number. **e**–**g**, Illustrations of the most recent *AMY2A* gene duplication, the complete loss of the *AMY2A* gene, and the sequential and joint duplication of *AMY2A* and *AMY2B* genes (grey shaded area in panel **c**). Blue, red and orange shaded areas indicate duplication, deletion and inversion events, respectively.

**Extended Data Fig. 3:**
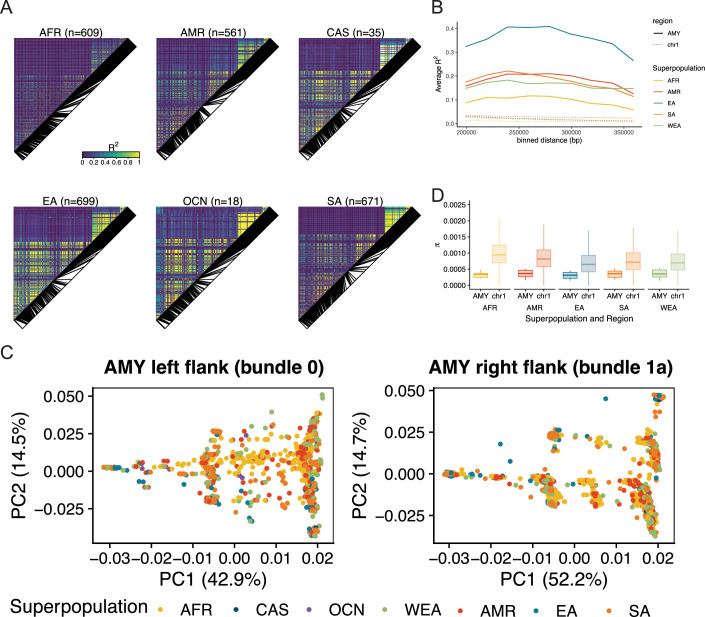
LD in different populations worldwide including 3,395 diverse diploid human genomes. **A**) Heat maps of linkage disequilibrium (LD) for SNPs across a ~ 406 kb region spanning unique sequences on either side of the structurally variable region of amylase (SVR) in different populations from seven continental regions (Africa - AFR, America - AMR, Central Asia - CAS, East Asia - EA, Oceania - OCN, South Asia - SA and Western Eurasia - WEA). **B**) LD decay over genomic distances for groups with more than 100 samples, measured as the average R2 between SNP pairs on either side of the SVR (i.e. 190 kb − 370 kb apart) binned into intervals of 20,000 bp, compared to identically spaced SNPs in chromosome 1. **C**) PCAs for non-duplicated regions adjacent to the SVR according to different continental regions using the distal (bundle 0) and proximal (bundle 1a) regions (see also Supplementary Fig. 5). **D**) Boxplots comparing π calculated in 20 kbp sliding windows across the distal non-duplicated region adjacent to the SVR for major continental human populations with more than 100 individuals. Centerline of the boxplot indicates the median, box limits indicate first and third quartiles, and whiskers indicate smallest/largest observation within box limits +/− 1.5 * interquartile range.

To discern the evolutionary origins of the vast diversity of structures observed, we sought to explore the SNP haplotypes on which they emerged. We leveraged unique sequences (bundles 0 and 1) flanking the SVR in which SNPs can be accurately genotyped. We first quantified linkage disequilibrium around the amylase locus in 3,395 diverse human samples (see Methods). To our surprise, linkage disequilibrium was extremely high between SNPs spanning the SVR (approximately 190–370 kb apart in GRCh38; Fig. 3a and Extended Data Fig. 3a,b). Of note, linkage disequilibrium was 7–20-fold higher than similarly spaced pairs of SNPs across the remainder of chromosome 1 in all major continental populations (Fig. 3b). Trio-based recombination rate estimates also indicate reduced recombination rates across the SVR^26^ (Fig. 3a, bottom panel). We hypothesize that these exceptionally high levels of linkage disequilibrium arise from the suppression of crossovers between homologues containing distinct structural architectures with vastly different lengths during meiosis.

**Extended Data Fig. 4:**
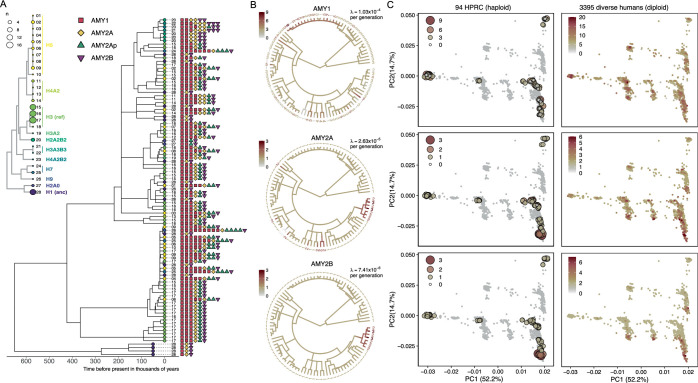
Reconstruction of the evolutionary history of amylase structural haplotypes using proximal unique sequence. **A**) A time-calibrated coalescent tree from the proximal non-duplicated region flanking the SVR (rightmost gray arrow in A until the recombination hotspot) across 94 assembled haplotypes (tree from the distal region in Fig. 3). The number next to each tip corresponds to the structural haplotype that the sequence is physically linked to and the color of the circle at each tip corresponds to its consensus haplotype structure (see inset structure tree). The copy numbers of each amylase gene and pseudogene are also shown next to the tips of the tree. **B**) Ancestral state reconstruction and mutation rate estimates for amylase gene copy number (archaic outgroups excluded). Branch color corresponds to copy number. **C**) A PCA from 94 haplotype assemblies and 3,395 diverse diploid human genomes from the proximal non-duplicated region flanking the SVR (PCA from the distal region in Extended Data Fig. 6). In the left column diploid genomes are shown in gray while assembled haplotypes are colored and sized by their haploid copy number of* AMY1*, *AMY2A*, and *AMY2B* genes. In the right column assembled haplotypes are hidden and diploid genomes are colored by their diploid copy number. As expected, clusters of diploid individuals with high copy number (right panels) tended to colocalize with assembled haplotypes containing duplications (left panels). Exceptions to this indicate heterozygotes (with placements in between two haplotypes) or additional duplication/deletion events.

The high linkage disequilibrium across the amylase locus implies that the evolutionary history of the flanking regions are a good proxy for the history of the linked complex structures of the SVR. As such, we constructed a maximum-likelihood coalescent tree from these blocks using three Neanderthal haplotypes and a Denisovan haplotype (all containing the ancestral structural haplotype) as outgroups (see Methods; Fig. 3c, Extended Data Fig. 4a and Supplementary Fig. 3). Time calibration of the tree was performed using an estimated 650 kyr bp human–Neanderthal split time^27^. Annotating this coalescent tree with the different amylase structural architectures revealed that most haplotype structures have experienced repeated evolution, where similar and even identical structures have arisen recurrently on different haplotype backgrounds. Only a handful of structural haplotypes are exceptions to this recurrence, including those with *AMY2B* gene duplications, which stem from a single originating haplotype.

**Extended Data Fig. 5:**
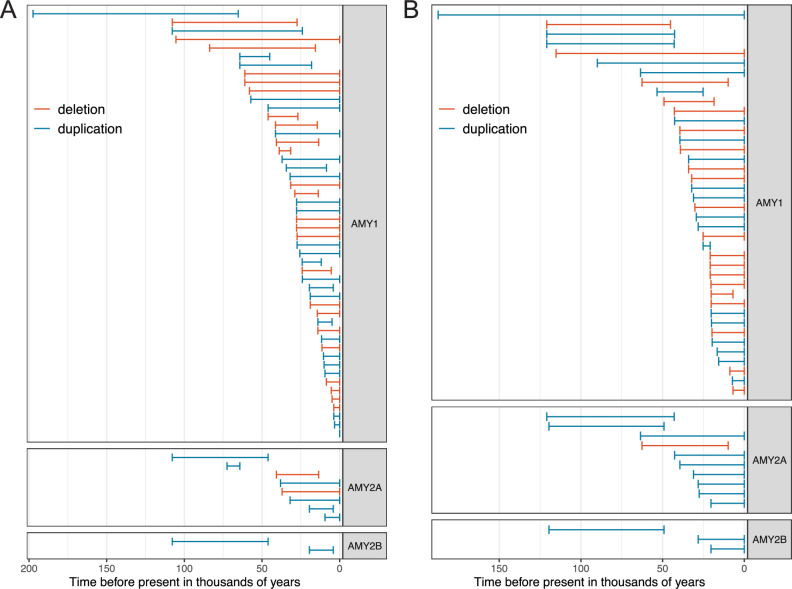
Estimated timings of amylase gene duplication and deletion events. These estimates are based on timed-calibrated coalescent trees using **A**) the bundle 0 sequence and **B**) the bundle 1a sequence.

Our time-calibrated tree further enabled us to perform an ancestral state reconstruction for each of the amylase gene copy numbers to quantify the number of times each gene has undergone duplication or deletion (Fig. 3d and Extended Data Figs. 4b and 5). We found that all amylase structural haplotypes in modern humans are descended from an H3^r^ haplotype approximately 279 kyr bp. This suggests that the initial duplication event, from the ancestral H1^a^ haplotype to H3^r^, significantly predates the out-of-Africa expansion (that is, more than 279 kyr bp). We identified 26 unique *AMY1* gene duplications and 24 deletions since then, corresponding to a per generation mutation rate (λ) of 2.09 × 10^−4^. Although these estimates may be affected by rare recombination events or additional unsampled duplications/deletions, their magnitude highlights the exceptional turnover of this locus in recent evolution, with *AMY1* gene copy number changes occurring at a rate of approximately 10,000-fold the genome-wide average SNP mutation rate^28^. *AMY2A* exhibited substantially fewer mutational events, undergoing six duplications and two deletions (λ = 3.07 × 10^−5^), with the most recent *AMY2A* duplication occurring within the past 9.4 kyr bp (Fig. 3c–e). Although duplications of *AMY2A* have occurred several times, we identified a single origin of the complete loss of the *AMY2A* gene in our tree, which occurred 13.5–40.7 kyr bp and resulted in the H2A0 haplotype (Fig. 3c,d,f). Only two *AMY2B* duplications were identified (λ = 7.36 × 10^−6^), occurring sequentially on a single haplotype and thus allowing us to resolve the stepwise process of their formation (Fig. 3c,d,g). We estimate that the first duplication event occurred 46–107.8 kyr bp, followed by a deletion 26.9–46 kyr bp, and finally by a second duplication event 4.1–19.5 kyr bp (Fig. 3g).

**Extended Data Fig. 6:**
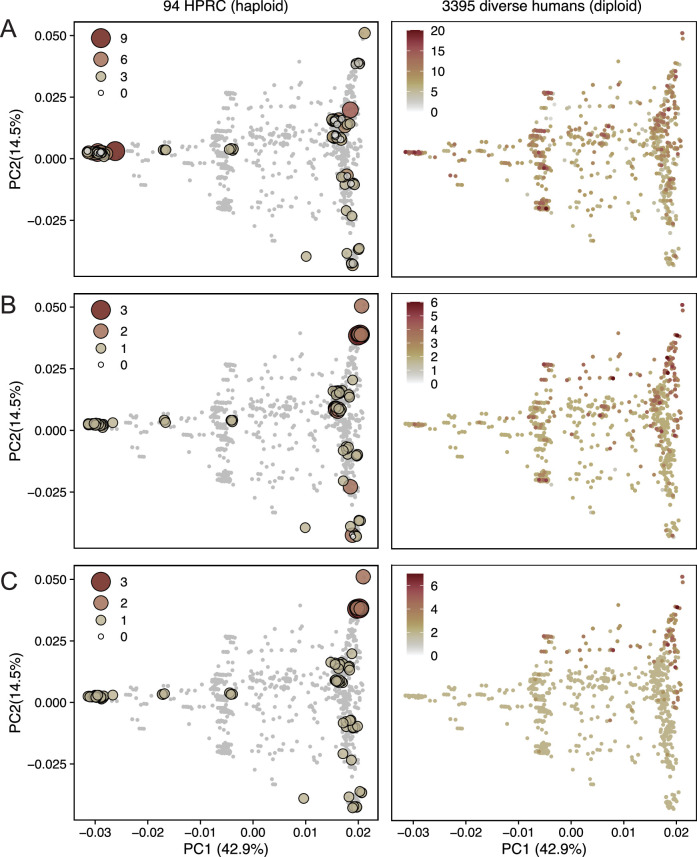
A PCA from 94 haplotype assemblies and 3,395 diverse diploid human genomes from the distal non-duplicated region flanking the SVR. In the left column diploid genomes are shown in gray while assembled haplotypes are colored and sized by their haploid copy number of **A**) *AMY1*, **B**)* AMY2A*, and **C**) *AMY2B*. In the right column assembled haplotypes are hidden and diploid genomes are colored by their diploid copy number. PCA from the proximal region in Extended Data Fig. 4c.

Although our collection of 94 assembled haplotypes spanning the complex SVR provides the most complete picture of amylase evolution to date, it still represents just a small fraction of worldwide genetic variation. To characterize the evolution of amylase haplotypes more broadly, we performed a principal component analysis combining the fully assembled haplotypes with 3,395 diverse human genomes using the flanking regions of the SVR (see Methods for details; Extended Data Figs. 3c, 4c and 6 and Supplementary Figs. 4 and 5). This method identified several additional *AMY1* and *AMY2A* duplication events worldwide, as expected given their high mutation rate, and support for additional haplotypes with complete *AMY2A* deletions (Extended Data Figs. 4c and 6 and Supplementary Fig. 4). However, we found no evidence of additional *AMY2B* gene duplications, supporting the single origin of these haplotypes.

## Pangenome-based haplotype deconvolution

Our analyses of SNP diversity at regions flanking the amylase SVR also revealed a substantial reduction in diversity compared with the chromosome-wide average (quantified by *π*, 2–3-fold lower; Extended Data Fig. 3d). To further investigate whether this signature was indicative of a selective sweep, we ran several genome-wide selection scans (Supplementary Table 5 and Supplementary Figs. 6–18). We found that some statistics tended to be higher at regions flanking the amylase SVR in specific populations (West Eurasians, Central Asia and Siberia and modern populations with traditionally agricultural diets; Supplementary Figs. 7, 9, 12 and 14), consistent with a soft or incomplete sweep. However, these results fell below the 99.95% threshold of the genome-wide empirical distribution, although this could be a consequence of the limitations of SNP-based methods in detecting selection at rapidly evolving, structurally complex loci, where identical structures repeatedly emerge on distinct haplotype backgrounds.

**Fig. 4:**
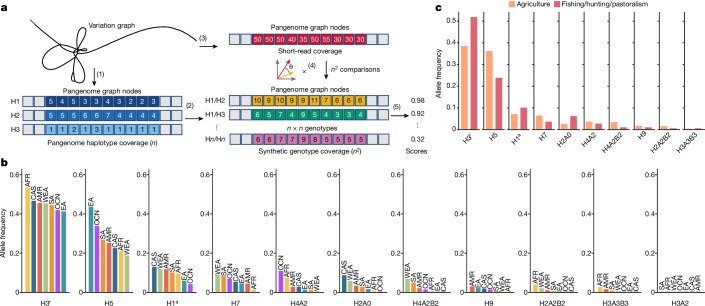
Inference of complex structural haplotypes from short-read data. **a**, Schematic of the haplotype deconvolution approach to infer the pair of structural haplotypes present in a short-read-sequenced individual. A set of assembled haplotypes are mapped to a variation graph, and coverage vectors are quantified over all nodes of the graph (1). Synthetic genotype vectors are constructed from summing all pairs of haplotype vectors (2). A short-read genome is mapped to the variation graph, and the read depth is quantified over all nodes in the graph (3). The short-read coverage vector is compared with all synthetic genotype vectors (4) and scored to identify the most likely haplotype pair present in the short-read-sequenced individual (5). **b**, Structural haplotype frequencies across continental populations in 3,594 diverse humans (7,188 haplotypes). **c**, Haplotype (allele) frequencies in individuals with traditionally agricultural subsistence compared with fishing-based, hunting-based and pastoralism-based diets.

**Extended Data Fig. 7:**
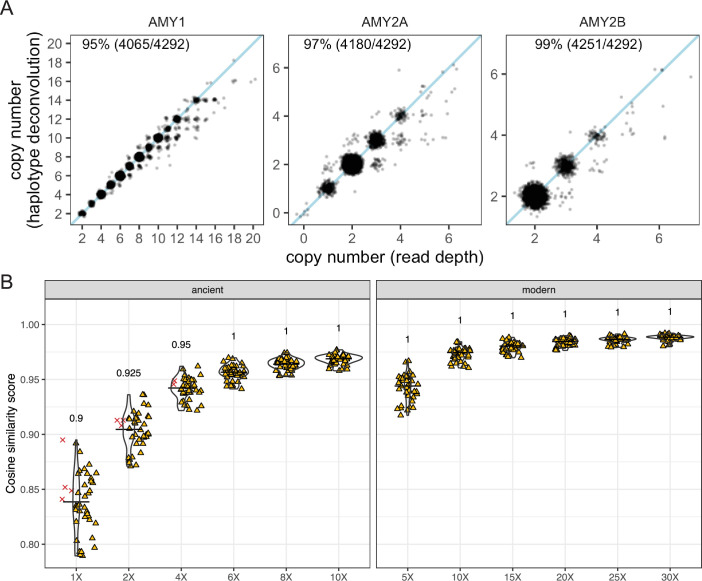
Validation of the haplotype deconvolution approach. **A**) Comparison between copy number estimates from read depth (x-axis) and copy number estimates from haplotype deconvolutions. Haplotype deconvolution copy number estimates come from the sum of the total copies of each gene on both haplotypes. The percentage indicated the number of exact matches between these two approaches. Points are jittered for visualization. **B**) Accuracy of haplotype deconvolution predictions on simulated ancient and modern genomes. Accuracy assessment of haplotype deconvolution predictions for both modern and ancient simulated genomes across varying coverage levels. Each data point represents a specific test, with the y-axis denoting the cosine similarity score. Red crosses indicate incorrect predictions, while yellow triangles represent correct predictions. The numbers atop each violin plot indicate the proportion of correct predictions (yellow triangles) within corresponding coverage groups.

Instead of relying on neighbouring SNPs as a proxy for amylase structural variants, we developed an approach to directly identify the structural haplotype pairs present in short-read-sequenced individuals. In brief, this approach, which we term ‘haplotype deconvolution’, consists of mapping a short-read-sequenced genome to the pangenome variation graph (Fig. 4a) and quantifying read depth over each node in the graph (*n* = 6,640 nodes in the amylase graph). This vector of read depths is then compared with a set of pre-computed vectors generated by threading all pairs of 94 long-read-assembled haplotypes (that is, all possible genotypes) over the same graph. Finally, we inferred the structural genotype of the short-read genome to be the pair of long-read-assembled haplotypes whose vector representation most closely matches to the short-read vector (see Methods). We assessed the accuracy of this approach using four orthogonal approaches (see Methods for details; Extended Data Fig. 7a). Together, these approaches indicate that our haplotype deconvolution method is robust and approximately 95% accurate, and limited primarily by the completeness of the reference pangenome.

We used haplotype deconvolution to estimate worldwide allele frequencies and continental subpopulation allele frequencies for amylase consensus structures across 7,188 haplotypes (Fig. 4b and Supplementary Tables 6 and 7). The reference haplotype, H3^r^, was the most common globally; however, several haplotypes exhibited strong population stratification. The H5 haplotype is the most frequent haplotype in East Asian populations, whereas the ancestral haplotype H1^a^ was underrepresented in East Asian and Oceanic populations. The high copy H9 haplotype was largely absent from African, West Eurasian and South Asian populations, whereas ranging from 1% to 3% in populations from the Americas, East Asia, and Central Asia and Siberia. Haplotypes with *AMY2B* duplications (that is, H2A2B2, H3A3B3 and H4A2B2) were essentially absent from East and Central Asia, explaining our previous observation of the lack of *AMY2B* duplication genotypes in these global populations (Fig. 1c) and consistent with their single origin.

We next compared the relative haplotype frequencies among modern human populations with traditionally agricultural-based, hunter-gatherer-based, fishing-based or pastoralism-based diets (Fig. 4c). Agricultural populations differed significantly from non-agricultural populations (*P* = 0.011, chi-squared test) and were enriched for haplotypes with higher *AMY1* copy number, including the H5, H7 and H9 haplotypes, as well as for haplotypes with higher *AMY2A* and *AMY2B* copy number (H4A2B2 and H2A2B2). By contrast, fishing-based, hunting-based and pastoralism-based populations were enriched for the reference H3^r^, deletion H2A0 and ancestral H1^a^ haplotypes. These results demonstrate that haplotypes with increased amylase gene copy number are enriched in modern-day populations with traditionally agricultural diets.

## Recent selection in West Eurasia

**Fig. 5:**
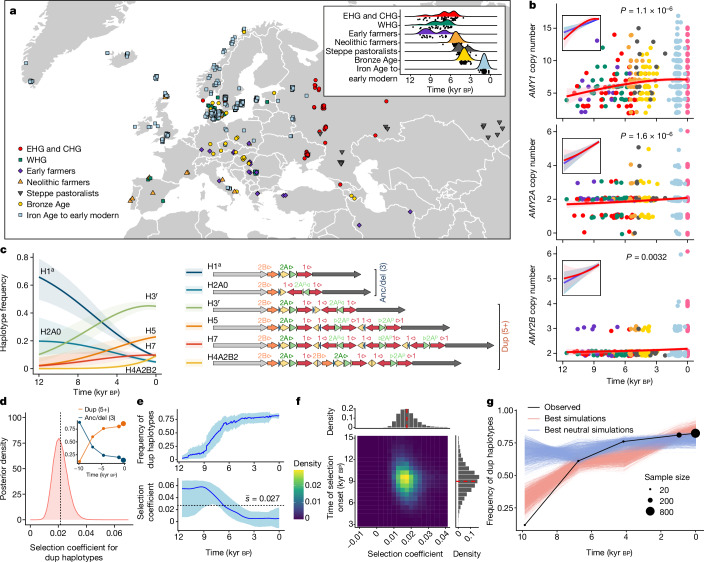
Recent selection at the amylase locus in West Eurasia. **a**, Locations of 533 West Eurasian ancient genomes from which amylase copy numbers were estimated. The inset shows the estimated ages of these samples. CHG, Caucasian hunter-gatherer; EHG, Eastern hunter-gatherer; WHG, Western hunter-gatherer. **b**, Copy number genotypes plotted as a function of age overlaid with a smooth generalized additive model fit. The inset shows the isolated linear model (blue) and the generalized additive model (red) fit to data. Two-sided *P* values from the linear model are shown without adjustment for multiple testing. The shaded areas indicate 95% confidence intervals of the fitted models. **c**, Haplotype trajectories fit by multinomial logistic regression for six haplotypes (right) present at more than 1% frequency in ancient and modern West Eurasians. The structures with the three total ancestral amylase copies (anc/del) are distinguished from duplication-containing haplotypes with five or more amylase genes (dup). The shaded areas indicate 95% confidence intervals. **d**, Posterior density of the selection coefficient for dup haplotypes over the past 12,000 years estimated from ApproxWF (mean of 0.022, indicated by the dotted line; no estimates ≤ 0 were observed in 1,000,000 MCMC iterations). The inset shows binned observations of dup versus anc/del haplotype frequency trajectories. **e**, Frequency and selection coefficient trajectories for dup haplotypes (blue line) and their 95% credible intervals (shaded area) estimated from bmws. **f**, Posterior distribution of the selection coefficient and the time of selection onset based on the ABC approach. The red dashed lines mark the median of the distribution. **g**, The observed allele frequency trajectory and the expected allele frequency trajectories from the top 1,000 of all simulations and the top 1,000 neutral simulations.

**Extended Data Fig. 8:**
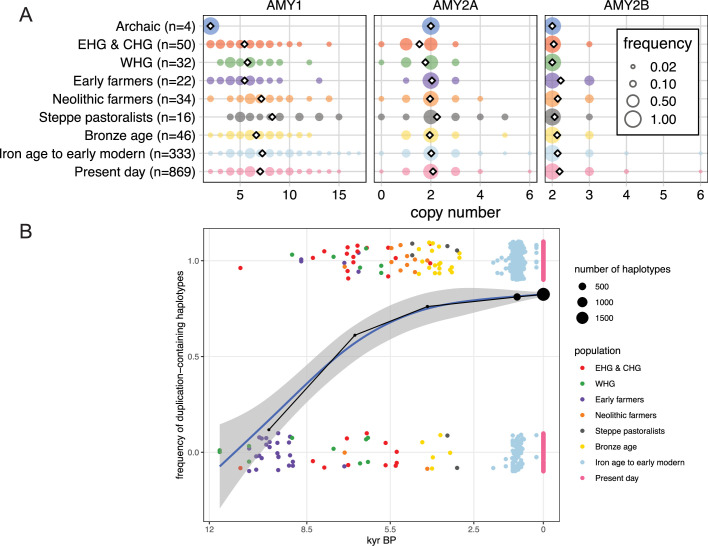
Structural diversity at the amylase locus in ancient samples in West Eurasia. **A**) The distribution of *AMY1*, *AMY2A*, and* AMY2B* copy numbers in ancient and modern populations of West Eurasia. The size of each point is proportional to the proportion of individuals in the population with that genotype. Diamonds indicate the population mean. **B**) Frequency of duplication-containing haplotypes over time. Each point represents a haplotype, and vertical jitter is added for legibility. A generalized additive model is fitted to the data, shown with the blue curve, and the 95% confidence interval is indicated by the shade. Binned haplotype frequency is shown with the black curve, with the following five time bins to maximize evenness in sample size across bins (unit in kyr BP): [12, 8.5), [8.5, 5.5), [5.5, 2.5), [2.5, 0), 0. This binned frequency trajectory is used as the summary statistics for the ABC analysis.

The development of agriculture approximately 12,000 years ago in the Fertile Crescent catalysed a rapid shift in the diets and lifestyles of West Eurasian populations. Most of the ancient genome sampling to date has been performed in Europe, allowing us to deeply explore the evolution of the amylase locus in these populations following the adoption of agriculture. To uncover how the genetic diversity of the amylase locus was shaped over this time period, we collated 533 recently generated ancient genomes from West Eurasia^29,30^, which span in age from approximately 12,000 to approximately 250 bp (Fig. 5a, Supplementary Table 8 and Supplementary Fig. 19). We estimated amylase gene copy numbers from these ancient individuals and compared these with copy numbers in modern Europeans (Extended Data Fig. 8a, Supplementary Table 1 and Supplementary Fig. 20). Overall, copy numbers of all amylase genes tended to be lower in ancient hunter-gatherer populations than in Bronze Age through present-day European populations, although these comparisons are of varying statistical significance due to our limited sample size of some ancient populations (ANOVA followed by Tukey’s test; Extended Data Fig. 8a and Supplementary Table 9). We next assessed how total copy numbers have changed as a function of time for each of the three amylase genes (Fig. 5b). In all three cases, we observed significant increases in total copy number over the past approximately 12,000 years (*P* = 1.1 × 10^−6^, *P* = 1.6 × 10^−6^ and *P* = 0.0032 for *AMY1*, *AMY2A* and *AMY2B*, respectively, linear model). The total *AMY1* copy number increased by an average of approximately 2.9 copies over this time period, whereas *AMY2A* and *AMY2B* increased by an average of 0.4 and 0.1 copies, respectively. These results are suggestive of directional selection at this locus for increased copy number of each of the three amylase genes.

We next applied our haplotype deconvolution approach to these ancient genomes to infer how the frequency of amylase structural haplotypes has changed over recent time. Simulations confirmed this method to be highly accurate even on low-coverage ancient genomes (see Methods; Extended Data Fig. 7b). We further conservatively selected 288 of the 533 individuals with the highest confidence haplotype assignments (see Methods; Supplementary Table 6 and Supplementary Figs. 21 and 22). Six haplotypes were found at appreciable frequencies (more than 1%) in either modern or ancient West Eurasian populations including the H1^a^ and H2A0 (*AMY2A* deletion) haplotypes, which each contain three total functional amylase gene copies, and the H3^r^, H5, H7 and H4A2B2 haplotypes, which contain between five and nine total amylase gene copies (Fig. 5c and Supplementary Fig. 23). Modelling the frequency trajectories of each of these haplotypes using multinomial logistic regression, we found that the ancestral H1^a^ and the H2A0 haplotypes both decreased significantly in frequency over the past approximately 12,000 years, from a combined frequency of approximately 0.88 to a modern-day frequency of approximately 0.14 (Fig. 5c,d, inset, Extended Data Fig. 8b and Supplementary Figs. 22 and 23). By contrast, duplication-containing haplotypes (with five or more amylase gene copies in contrast to the ancestral three copies; note that no haplotypes containing four copies were observed) increased in frequency commensurately more than sevenfold (from approximately 0.12 to approximately 0.86) over this time period.

We used three complementary approaches to test whether positive selection could explain the substantial rise in the frequency of duplication-containing haplotypes (see Methods for model parameters and assumptions). First, we used a Bayesian approach that assumes a constant population size and selection coefficient (ApproxWF^31^). The posterior distribution of the selection coefficient (*s*) supported positive selection (*P* < 1 × 10^−6^, empirical *P* value) with an average of *s*_dup_ = 0.022 (Fig. 5d). We next used bmws^32^, which allows *s*_dup_ to vary over time. Selection was found to be the strongest 12–9 kyr bp, with *s*_dup_ approaching 0.06 (Fig. 5e). Subsequently, selection has significantly weakened, approaching 0 in recent times (average *s*_dup_ = 0.027; Fig. 5e). Finally, we implemented an approximate Bayesian computation approach adapted and modified from Kerner et al.^33^ to account for the important demographic factors that shape allele frequencies over time (for example, population structure, admixture events and population growth; see Methods). The posterior distribution of *s*_dup_ is centred around 0.0175 and does not overlap 0, whereas the time of the selection onset is estimated to be around 9 kyr bp (Fig. 5f and Supplementary Fig. 24). In addition, none of the neutral simulations conducted (that is, with *s*_dup_ = 0) exhibits higher allele frequency increases than observed in the data (Fig. 5g and Supplementary Fig. 25). Together, these results are consistent with positive selection for duplication-containing haplotypes at the amylase locus following the adoption and spread of agriculture in West Eurasia.

## Discussion

The domestication of crops and subsequent rise of farming radically reshaped human social structures, lifestyles and diets. Several evolutionary signatures of this transition have been identified in ancient and modern West Eurasian genomes^30,34,35^. However, although it has been hypothesized that the amylase locus has similarly undergone selection due to this transition^2^, footprints of recent positive selection have not been detected to date^3,4^. Here, taking advantage of long-read assemblies, we characterized the complex haplotype structures at the amylase locus to the highest resolution to date, illuminating structural and sequence complexity intractable to short-read sequencing (for example, Supplementary Fig. 26). Furthermore, these long-read haplotypes provide previously inaccessible information about flanking SNPs linked to these complex structures. These enable us to build coalescent trees revealing the rapid and repeated duplication and deletion events at this locus in recent human history. In particular, we found that the majority of these events occurred within the past 50 kyr and thus would only be tagged by rare variants in the flanking region. Thus, the extensive homoplasy and high mutation rate at this region make flanking SNPs poor tags in classical tests for selective sweeps^36,37^, potentially explaining the failure of previous efforts aimed at detecting selection at this locus. Finally, we leveraged long-read assemblies to improve the utility of existing short-read data by constructing pangenome graphs of the amylase locus, which we used to infer the haplotype structure in short-read-sequenced individuals. This graph-based approach, termed haplotype deconvolution, unlocks the ability for regions previously inaccessible to short reads to now be revisited in both modern and ancient datasets.

Using our haplotype deconvolution approach, we were able to confidently reconstruct the haplotype structures of 288 ancient samples at the amylase locus. We found that haplotypes carrying duplicated copies of amylase genes have increased in frequency sevenfold in the past 12,000 years. We note that our analyses are limited by the relatively low sample sizes and uneven sampling of high-quality ancient genomes in West Eurasia that are suitable for haplotype assignment. The several approaches that we used to test for selection are also dependent on various model assumptions and genotyping accuracy. Nevertheless, we present multiple lines of evidence (Figs. 1d, 4c and 5b–g) that consistently support recent selection in West Eurasians at the amylase locus potentially linked to the adoption of agriculture.

One of the best-studied examples of human adaptation to diet is the evolution of lactase persistence^5,6^ (although see refs. ^38,39^ regarding potential complexities underlying selection at this locus). Our estimates of *s*_dup_ are comparable in magnitude to estimates of *s* at the *MCM6/LCT* locus reported in many studies^32,33,38^. However, increased *AMY1* copy numbers have also been associated with deleterious oral health outcomes^40^ (that is, cavities), highlighting a potential evolutionary trade-off, which might result in distinct selection dynamics in contrast to other diet-associated loci such as *LCT*. The repeated mutation and homoplasy found at the amylase locus adds further evolutionary complexity, in contrast to loci driven by point mutations. We found the mutation rate of amylase gene duplications/deletions to be approximately 10,000-fold the average SNP mutation rate, similar to short tandem repeats^41^. This is similar to recently described structural variation mutation rates at ampliconic Y chromosome regions^42^. In both cases, the duplication architecture of the locus potentially predisposes to de novo structural variant formation through non-allelic homologous recombination between long paralogous sequences on the same chromatid or sister chromatids^43^, or non-crossover gene conversion, which can yield similar structural variants^44^. Thus, linkage disequilibrium is maintained across the locus, even in the presence of rapid, recurrent structural changes.

Another interesting parallel between *MCM6/LCT* and the amylase locus is that the ability to digest milk has arisen independently in different populations^5,6^. Similarly, agriculture has been adopted independently several times throughout human history^1^. Here, in addition to showing evidence of positive selection in West Eurasian populations, we found that haplotypes carrying higher amylase copy numbers are found more commonly in multiple other populations with traditionally agricultural subsistence worldwide. These results suggest that selection for increased amylase copy number may have also happened several times throughout human history, coincident with the several independent adoptions of agriculture. Because ancient samples from regions other than Europe are scarce, we were not able to infer potential selection associated with other agricultural adoptions. More extensive sampling of diverse ancient genomes and modern long-read assemblies are needed to further test this hypothesis. The expansion of amylase genes accompanying transitions to starch-rich diets appears to have also occurred independently across several different commensal species including dogs, pigs, rats and mice, highlighting the repeated evolution of this locus across taxa^12,45^ and the far-reaching effect of the agricultural revolution on the genetics and evolution of species beyond our own.

## Methods

### Amylase gene naming conventions

The reference genome GRC38 represents an H3 haplotype with three copies of the *AMY1* gene and one copy each of the *AMY2A* and *AMY2B* genes. The three *AMY1* copies are identified with labels *AMY1A*, *AMY1B* and *AMY1C* due to the HUGO naming convention requirements for all gene copies to have unique names. However, these various copies of *AMY1* genes across different haplotypes are recent duplications that share high sequence similarity, and therefore are referred to simply as *AMY1* genes in this paper and others^2,3,15^. By contrast, *AMY2A* and *AMY2B* stem from a much older gene duplication event and are much more diverged than the different copies of *AMY1* genes^13^. They share the AMY2 prefix simply because they are both expressed in the pancreas.

### Datasets

Short-read sequencing data were compiled from high-coverage resequencing of the 1,000 Genomes Project (1KG) samples^19^, the Simons Genome Diversity Panel (SGDP)^20^, and the Human Genome Diversity Panel (HGDP)^18^. Genomes from GTEx^21^ samples were also assessed, but only for gene expression analyses as the ancestry of these samples was not available. In total, we obtained copy number genotype estimates for 5,130 contemporary samples. Among these, 838 are GTEx samples, 698 are trios from the 1KG, and the rest (*n* = 3,594, that is, 7,188 haplotypes) are unrelated individual samples compiled from the 1KG, HGDP and SGDP. GTEx and 1KG trio samples were excluded from analyses characterizing the global diversity of the amylase locus. We performed haplotype deconvolutions on all unrelated samples as well as trio data (*n* = 4,292 total), but the trios were only used for validation purposes.

Supplementary Fig. 25 shows structural variant calls from the gnomAD project^46^. Phased SNP calls from 1KG and HGDP samples were compiled from Koenig et al.^47^, which includes all of our 1KG and HGDP samples but only some of the SGDP samples (*n* = 3,395 total). These data were used for the analyses of linkage disequilibrium, nucleotide diversity, principal component analysis (PCA) and selection scans^47^.

Ancient genome short-read fastq samples were compiled from Allentoft et al.^30^ and Marchi et al.^29^ and were mapped to the human reference genome GRCh38 with BWA (v0.7.17; ‘bwa mem’)^48^. The modern genomes and the 14 Marchi et al. genomes are of high coverage and quality; however, the Allentoft et al. samples were of varying quality and coverage^30^. The Allentoft et al. dataset included more than 1,600 ancient genomes including 317 newly sequenced ancient individuals alongside 1,492 previously published genomes. Unfortunately, many published ancient genomes have been filtered to exclude multi-mapped reads leaving large gaps over regions such as the amylase locus. After removing genomes with missing data, 690 samples remained. We carefully analysed these 690 genomes to determine their quality by quantifying the standard deviation of genome-wide copy number (after removing the top and bottom fifth percentiles of copy number to exclude outliers). We chose a standard deviation cut-off of 0.49 based on a visual inspection of the copy number data and selected 519 samples (approximately 75% of 690) with sufficient read depth for copy number genotyping. Ancient samples were assigned to one of eight major ancient populations in West Eurasia based on their genetic ancestry, location and age obtained from their original publications^29,30,49,50^ (Fig. 5a, Supplementary Table 8 and Supplementary Fig. 19). These populations include: Eastern hunter-gatherer, Caucasian hunter-gatherer, Western hunter-gatherer, early farmer (samples with primarily Anatolian farmer ancestry), Neolithic farmer (samples with mixed Anatolian farmer and Western hunter-gatherer ancestry), Steppe pastoralist (samples with mixed Eastern hunter-gatherer and Caucasian hunter-gatherer ancestry), Bronze Age (samples with mixed Neolithic farmer and Steppe ancestry), and Iron Age to early modern. Finally, four archaic genomes were assessed including three high-coverage Neanderthal genomes and the high-coverage Denisova genome^27,51–53^.

Long-read haplotype assemblies were compiled from the HPRC^23^. Year 1 genome assembly freeze data were compiled along with year 2 test assemblies. Haplotype assemblies were included in our analyses only if they spanned the amylase SVR. Furthermore, in cases in which both haplotypes of an individual spanned the SVR, we checked to ensure that the diploid copy number of amylase genes matched with the read-depth-based estimate of copy number. We noted that several year 1 assemblies (which were not assembled using ONT ultralong sequencing data) appeared to have been misassembled across the amylase locus, as they were either discontiguous across the SVR or had diploid assembly copy numbers that did not match with short-read-predicted copy number. We thus reassembled these genomes incorporating ONT ultralong sequence using the Verkko assembler (v1.3.1)^54^, constructing improved assemblies for HG00673, HG01106, HG01361, HG01175, HG02148 and HG02257. Alongside these HPRC genome assemblies, we included GRCh38 and the newly sequenced T2T-CHM13 reference^24^.

### Determination of subsistence by population

The diets of several populations (see Supplementary Table 2) were determined from the literature from the following sources^2,55–63^. We were able to identify the traditional diets for 33 populations. All other populations were excluded from this analysis.

### Read-depth-based copy number genotyping

Copy number genotypes were estimated using read depth as described in ref. ^16^. In brief, read depth was quantified from BAMs in 1,000-bp sliding windows in 200-bp steps across the genome. These depths were then normalized to a control region in which no evidence of copy number variation was observed in more than 4,000 individuals. Depth-based ‘raw’ estimates of copy number were then calculated by averaging these estimates over regions of interest. Regions used for genotyping are found in Supplementary Table 10. We note that the *AMY2Ap* pseudogene is a partial duplication of *AMY2A* that excludes the approximately 4,500 bp of the 5′ end of the gene. This region can thus be used to genotype the *AMY2A* copy without ‘double counting’ *AMY2Ap* gene duplicates. Copy number genotype likelihoods were estimated by fitting modified Gaussian mixture model to raw copy estimates across all individuals with the following parameters: *k*, the number of mixture components, set to be the difference between the highest and lowest integer-value copy numbers observed; *π*, a *k*-dimensional vector of mixture weights; σ, a single-variance term for mixture components; and *o*, an offset term by which the means of all mixture components are shifted. The difference between mixture component means was fixed at 1, and the model was fit using expectation maximization (Supplementary Fig. 1). The copy number maximizing the likelihood function was used as the estimated copy number for each individual in subsequent analyses. Comparing these maximum likelihood copy number estimates with droplet digital PCR yielded very high concordance with *r*^2^ = 0.98, 0.99 and 0.96 for *AMY1*, *AMY2A* and *AMY2B*, respectively (Supplementary Fig. 1). For comparisons of copy number as a function of sustenance, populations were downsampled to a maximum of 50 individuals. We also used a linear mixed effects model approach in which all samples were maintained, which provided similar results (*P* = 0.013, *P* = 0.058 and *P* = 0.684 for *AMY1*, *AMY2A* and *AMY2B*, respectively).

### Analysis of gene expression

Gene expression data from the GTEx project^21^ were downloaded alongside short-read data (see above section). Normalized gene expression values for *AMY2A* and *AMY2B* were compared with copy number estimates using linear regression (Extended Data Fig. 2).

### MAP-graph construction

Regions overlapping the amylase locus were extracted from genome assemblies in two different ways. First, we constructed a PanGenome Research Tool Kit (PGR-TK) database from the HPRC year 1 genome assemblies and used the default parameters of *w* = 80, *k* = 56, *r* = 4 and min-span = 64 for building the sequence database index. The GRCh38 chromosome 1: 103655518–103664551 was then used to identify corresponding *AMY1*/*AMY2A*/*AMY2B* regions across these individuals. Additional assemblies were subsequently added to our analysis by using minimap2 (ref. ^64^) to extract the amylase locus from those genome assemblies. The MAP-graph and the principal bundles were generated using revision (v0.4.0; git commit hash: ed55d6a8). The Python scripts and the parameters used for generating the principal bundle decomposition can be found in the associated GitHub repository. The position of genes along haplotypes was determined by mapping gene modes to haplotypes using minimap2 (ref. ^64^).

### Analysis of mutations at amylase genes

To identify mutations in amylase genes from long-read assemblies and evaluate their functional impact, we first aligned all amylase gene sequences to *AMY1A*, *AMY2A* and *AMY2B* sequences on GRCh38 using minimap2 (ref. ^64^). We then used paftools.js^64^ for variant calling, and vep-v.105.0 (ref. ^65^) for variant effect prediction.

### PGGB-based graph construction

Although the existing pangenome graphs from the HPRC provide a valuable resource, we discovered that they did not provide the best reference system for genotyping copy number variation. Our validation of the genotyping approach revealed that we would experience high genotyping error when gene copies (for example, all copies of *AMY1* or all copies of *AMY2B*) were not fully ‘collapsed’ into a single region in the graph. We thus elected to rebuild the graph locally to improve genotyping accuracy for complex structural variants. This achieves substantially improved results by allowing multiple mappings of each haplotype against others, which leads to a graph in which multi-copy genes are collapsed into single regions of the graph. This collapsed representation is important for graph-based genotyping. In addition, we incorporated additional samples, some of which were reassembled by us, that were not part of the original dataset from the HPRC to have a more comprehensive representation of variability in the amylase locus, which required rebuilding the pangenome graph model at the amylase locus.

A PGGB graph was constructed from 94 haplotypes spanning the amylase locus using PGGB (v0.5.4; commit 736c50d8e32455cc25db19d119141903f2613a63)^25^ with the following parameters: ‘-n 94’ (the number of haplotypes in the graph to be built) and ‘-c 2’ (the number of mappings for each sequence segment). The latter parameter allowed us to build a graph that correctly represents the high copy number variation in such a locus. We used ODGI (v0.8.3; commit de70fcdacb3fc06fd1d8c8d43c057a47fac0310b)^66^ to produce a Jaccard distance-based (that is, 1 − Jaccard similarity coefficient) dissimilarity matrix of paths in our variation graph (‘odgi similarity -d’). These pre-computed distances were used to construct a tree of relationships between haplotype structures using neighbour joining.

### Haplotype deconvolution approach

We implemented a pipeline based on the workflow language Snakemake (v7.32.3) to parallelize haplotype deconvolution (that is, assign to a short-read-sequenced individual the haplotype pair in a pangenome that best represents its genotype at a given locus) in thousands of samples.

Given a region-specific PGGB graph (gfa; see ‘PGGB-based graph construction’), a list of short-read alignments (BAM/CRAM), a reference build (fasta) and a corresponding region of interest (chr: start–end; based on the alignment of the BAM/CRAM), our pipeline ran as follows:

1. Extracted the haplotypes from the initial pangenome using ODGI (v0.8.3; ‘odgi paths -f’)^66^.
2. For each short-read sample, extracted all the reads spanning the region of interest using SAMTOOLS (v1.18; ‘samtools fasta’)^67^.
3. Mapped the extracted reads back to the haplotypes with BWA (v0.7.17; ‘bwa mem’)^48^. To map ancient samples, we used ‘bwa aln’ with parameters suggested in Oliva et al.^68^ instead: ‘bwa aln -l 1024 -n 0.01 -o 2’.
4. Computed a node depth matrix for all the haplotypes in the pangenome; every time a certain haplotype in the pangenome loops over a node, the path depth for that haplotype over that node increases by one. This was done using a combination of commands in ODGI (‘odgi chop -c 32’ and ‘odgi paths -H’).
5. Computed a node depth vector for each short-read sample; short-read alignments were mapped to the pangenome using GAFPACK (https://github.com/ekg/gafpack; commit ad31875) and their coverage over nodes was computed using GFAINJECT (https://github.com/ekg/gfainject; commit f5feb7b).
6. Compared each short-read vector (see step 5) with each possible pair of haplotype vectors (see step 4) by means of cosine similarity using (https://github.com/davidebolo1993/cosigt; commit e247261; which measures the similarity between two vectors as their dot product divided by the product of their lengths). The haplotype pair having the highest similarity with the short-read vector was used to describe the genotype of the sample.
7. The final genotypes were assigned as the corresponding consensus structures of the highest similarity pair of haplotypes.

Our pipeline is publicly available on GitHub (https://github.com/raveancic/graph_genotyper) and is archived in Zenodo (https://zenodo.org/doi/10.5281/zenodo.10843493).

We assessed the accuracy of the haplotype deconvolution approach in several different ways. First, we assessed 35 individuals (70 haplotypes) for which both short-read sequencing data and long-read diploid assemblies were available. In 100% of cases (70 of 70 haplotypes), we accurately distinguished the correct haplotypes present in an individual from short-read sequencing data. We further assessed how missing haplotypes in the pangenome graph might assess the accuracy of our approach by performing a ‘leave-one-out, jackknifing’ analysis. In this approach, for each of the 35 long-read individuals, we rebuilt the variation graph with a single haplotype excluded and tested our ability to identify the correct consensus haplotype from the remaining haplotypes. The true positive rate was approximately 93% in this case. Second, we compared our haplotype deconvolutions to haplotypes determined by inheritance patterns in 44 families in a previous study^15^ (Supplementary Table 3). We note that this study hypothesized the existence of an H4A4B4 haplotype without having observed it directly. In our study, we also found no direct evidence of the H4A4B4 haplotype. Furthermore, we found that inheritance patterns are equally well explained by other directly observed haplotypes and thus exclude these predictions from our comparisons (two individuals excluded). We identified the exact same pair of haplotypes in 95% of individuals (125 of 131 individuals), and in 97% of individuals (288 of 298 individuals), the haplotype pair that we identified is among the potential consistent haplotype pairs identified from inheritance. Third, we compared inheritance patterns in 602 diverse short-read-sequenced trios from the 1KG populations^19^. For each family, we randomly selected one parent and assessed whether either of the two offspring haplotypes were present in this randomly selected parent. Across all families, this proportion, *p*, represents an estimate of the proportion of genotype calls that are accurate in both the offspring and that parent, thus the single sample accuracy can be estimated as the square root of *p*. From these analyses, we identified 533 of 602 parent–offspring genotype calls that are correct, corresponding to an estimated accuracy of 94%. Fourth, we compared our previously estimated reference genome read-depth-based copy number genotypes to those predicted from haplotype deconvolutions across 4,292 diverse individuals. These genotypes exhibited 95–99% concordance across different amylase genes (95%, 97% and 99% for *AMY1*, *AMY2A* and *AMY2B*, respectively). Cases in which the two estimates differed were generally high-copy genotypes for which representative haplotype assemblies have not yet been observed and integrated into the graph (Extended Data Fig. 7a). Overall we thus estimated the haplotype deconvolution approach to be approximately 95% accurate for modern samples, and thus choose not to propagate the remaining 5% uncertainty into downstream analyses.

To determine the impact of coverage and technical artefacts common in ancient DNA, we performed simulations. We selected 40 individuals having both haplotypes represented in the AMY graph and, for those, we simulated short reads mirroring error profiles in modern and ancient genomes across different coverage levels. More specifically, we simulated paired-end short reads for the modern samples with wgsim (https://github.com/lh3/wgsim; commit a12da33, ‘wgsim −1 150 −2 150’) and single-end short reads for the ancient samples with NGSNGS^69^ (commit 559d552, ‘ngsngs -ne -lf Size_dist_sampling.txt -seq SE -m b7,0.024,0.36,0.68,0.0097 -q1 AccFreqL150R1.txt’ following the suggestions by the author in https://github.com/RAHenriksen/NGSNGS). Synthetic reads were then aligned against the GRCh38 build of the human reference genome using bwa-mem2 (ref. ^70^; commit 7f3a4db). For samples modelling modern individuals, we generated 5–30X coverage data, whereas for those modelling ancient genomes, we aimed for lower coverage (1–10X) to better approximate true-to-life data. We ran our haplotype deconvolution pipeline independently for modern and ancient simulated samples, as well as varying coverage levels. Out of 480 tests, only 9 (approximately 1%) yielded incorrect predictions, exclusively in ancient simulated sequences, with coverage ranging from 1X to 4X. Cosine similarity scores for ancient simulated sequences ranged from 0.789 to 0.977 (median of 0.950), whereas scores for modern simulated sequences ranged from 0.917 to 0.992 (median of 0.981; Extended Data Fig. 7b). We therefore conclude that the haplotype deconvolution method is also highly accurate for ancient samples. Out of an abundance of caution, we further imposed a conservative quality score threshold of 0.75 to ancient samples, resulting in 288 ancient samples with high-confidence haplotype assignment out of a total of 533 (Supplementary Figs. 20 and 21). We note that the haplotype deconvolutions in ancient samples are probably more accurate than read-depth genotypes, which tend to be biased towards higher copy number.

### Linkage disequilibrium estimation

To investigate pairwise linkage disequilibrium across the SVR region at a global scale, we first merged our copy number estimates with the joint SNP call set from the HGDP and 1KG^47^, resulting in a variant call set of 3,395 diverse individuals with both diploid copy number genotypes and phased SNP calls. In brief, we used bcftools (v1.9)^67^ to filter HGDP and 1KG variant data for designated genomic regions on chromosome 1, including the amylase SVR and flanking regions defined as bundle 0 and bundle 1 (distal and proximal, respectively) using the GRCh38 reference coordinate system (--region chromosome 1: 103,456,163–103,863,980 in GRCh38). The resulting output was saved in variant call format (vcf), keeping only biallelic SNPs (-m2 -M2 -v snps), and additionally filtered with vcftools (v.0.1.16)^71^ with -keep and -recode options for lists of individuals grouped by continental region in which we were able to estimate diploid copy numbers. Population-specific vcf files were further filtered for a minor allele frequency filter threshold of 5% (--minmaf 0.05) and used to generate a numeric genotype matrix with the physical positions of SNPs for linkage disequilibrium calculation (*R*^2^ statistic) and plotting with the LDheatmap^72^ function in R (v4.2.2).

To further dissect the unique evolutionary history of the amylase locus, we compared regions with high *R*^2^ across the SVR with linkage disequilibrium estimates for pairs of SNPs across regions of similar size in chromosome 1. We specifically focused on pairs of SNPs spanning bundle 0 (chromosome 1: 103456163–103561526 in GRCh38) and the first 66-kb of bundle 1, hereafter labelled as bundle 1a (chromosome 1: 103760698–103826698 in GRCh38), as revealed by the linkage disequilibrium heatmap. Then, we computed the *R*^2^ values for any pair of SNPs in chromosome 1 for each superpopulation within a minimum of 190-kb distance (that is, the equivalent distance from the bundle 0 end to the bundle 1a start using the GRCh38 reference coordinate system) and maximum 370-kb distance (that is, the equivalent distance from the bundle 0 start to the bundle 1a end using the GRCh38 reference coordinate system). To calculate pairwise linkage disequilibrium across the human chromosome 1 for different populations, we ran plink (v1.90b6.21)^73^ with options -r2 –ld-window 999999 –ld-window-kb 1000 –ld-window-r2 0 –make-bed –maf 0.05, using population-specific vcf files for a set of biallelic SNPs of 3,395 individuals from the HGDP and 1KG as input. As the resulting plink outputs only provide *R*^2^ estimates for each pair of SNPs and respective SNP positions, we additionally calculated the physical distances between pairs of SNPs as the absolute difference between the base-pair position of the second (BP_B) and first (BP_A) SNP. We then filtered out distances smaller than 190 kb and greater than 370 kb, and annotated the genomic region for each *R*^2^ value based on whether both SNPs fall across the SVR or elsewhere in chromosome 1. The distance between SNP pairs was also binned into intervals of 20,000 bp, and the midpoint of each interval was used for assessing linkage disequilibrium decay over genomic distances. The resulting dataset was imported in R to compute summary statistics comparing linkage disequilibrium across each major continental region, or superpopulations, and we used ggplot2 to visualize the results.

### Coalescent tree, ancestral-state reconstruction and PCA

To construct the coalescent tree, we first extracted bundle 0 and bundle 1a sequences from all 94 haplotypes (that is, distal and proximal unique regions flanking the amylase SVR) that went through principal bundle decomposition. On the basis of their coordinates on the human reference genome (GRCh38), we used SAMtools (v1.17)^74^ to extract these sequences from three Neanderthal and one Denisovan genomes that are aligned to GRCh38. We used kalign (v3.3.5)^75^ to perform multiple sequence alignment on bundle 0 and bundle 1a sequences. We used IQ-TREE (v2.2.2.3)^76^ to construct a maximum likelihood tree with Neanderthal and Denisova sequences as the outgroup, using an estimated 650 kyr human–Neanderthal split time for time calibration^27^. We used ggtree (v3.6.2)^77^ in R (v4.2.1) to visualize the tree and annotated each tip with its structural haplotype and amylase gene copy numbers. We used cafe (v5.0.0)^78^ to infer the ancestral copy numbers of each of the three amylase genes along the time-calibrated coalescent tree (excluding the outgroups) and to estimate their duplication/deletion rates. The timing of each duplication/deletion event was estimated based on the beginning and end of the branch along which the amylase gene copy number had changed. We used ggtree and ggplot (v3.4.2) in R to visualize these results, and used Adobe Illustrator (v27.5) to create illustrations for several of the most notable duplication/deletion events^79^.

Next, we performed a PCA combining 94 HPRC haplotype sequences with variant calls for 3,395 individuals from the HGDP and 1KG. We first aligned all 94 bundle 0 and 94 bundle 1a haplotype sequences to the human reference genome (GRCh38) using minimap2 (v2.26)^64^, and called SNPs from haplotypes using paftools.js. Each haplotype sequence appears as a pseudo-diploid in the resulting vcf file (that is, when the genotype is different from the reference, it is coded as being homozygous for the non-reference allele). These haplotype-specific vcf files were merged together and filtered for biallelic SNPs (-m2 -M2 -v snps) with bcftools, resulting in a pseudo-diploid vcf file from 94 haplotype sequences for each bundle. These were then merged with the respective bundle 0 and bundle 1a vcf files from the HGDP and 1KG, also filtered for biallelic SNPs, using bcftools. Finally, we ran plink with a minor allele frequency of 5% (--maf 0.05) to obtain eigenvalues and eigenvectors for PCA and used ggplot (v3.4.2) to visualize the results. These analyses were conducted with bundle 0 and bundle 1a separately, with highly concordant results (Supplementary Figs. 3 and 4). Analyses focused on bundle 0 are mostly reported in the main text (Fig. 3 and Extended Data Fig. 6), whereas bundle 1a results are shown as extended data (Extended Data Fig. 4).

### Signatures of recent positive selection in modern human populations

To investigate very recent or ongoing positive selection at the amylase locus in modern humans, we first looked for significant signatures of reduced genetic diversity across the non-duplicated regions adjacent to the SVR compared with chromosome 1 in different populations worldwide. This stems from the assumption that, given low SNP density across the SVR, the high levels of linkage disequilibrium found between pairs of SNPs spanning bundle 0 and bundle 1a indicate that SNPs in bundle 0 or bundle 1 can be used as proxies for the selective history of the linked complex structures of the SVR. We calculated nucleotide diversity (*π*) on sliding windows of 20,000 bp spanning GRCh38 chromosome 1 with vcftools using population-specific vcf files from the HGDP and 1KG filtered for a set of biallelic SNPs as input. Each window was annotated for the genomic region, namely, bundle 0, SVR and bundle 1a. All windows comprising the SVR were removed from the resulting output due to low SNP density. We then used ggplot2 in R to compare and visualize nucleotide diversity in the flanking regions of the amylase locus (that is, bundle 0 and bundle 1a) and the rest of chromosome 1 for each major continental region or super-population.

To identify either soft-selective and hard-selective sweeps at the flanking regions of the SVR, we computed several different extended haplotype homozygosity-based statistics and statistics based on distortions of the haplotype frequency spectrum (Supplementary Table 5). Vcf files from the HGDP and 1KG chromosomes 1–22 GRCh38 were filtered for biallelic SNPs and minor allele frequency of 0.05 for target populations with over 10 individuals to calculate iHS^80^, nSL^81^ and XP-nSL^82^ as implemented in selscan (v2.0.2)^83^ (see Supplementary Table 5 for a description of populations and selection statistics). Utah residents with Northern and Western European ancestry (CEU) and Yoruba (YRI) populations were also included to confirm the ability of the tests to consistently identify the *LCT* hard sweep in CEU and in relation to the amylase locus (Supplementary Table 5). Scores for these statistics were normalized using the genome-wide empirical background with selscan’s co-package norm (v1.3.0). This was also used to compute the fraction of the standardized absolute values > 2 for each statistic in non-overlapping 100-kb windows genome-wide^80^. For XP-nSL statistics, modern rainforest hunter-gatherers in Africa and the pastoralists Yakut were used as reference populations, so that positive scores correspond to possible sweeps in the populations with traditionally agricultural diets. We also used lassip (v1.2.0)^84^ to compute H12 and H2/H1 statistics^85^ and saltiLASSI Λ^84^ on sliding windows of 201 SNPs with intervals of 100 SNPs. SNP positions within the SVR were removed from the resulting outputs due to low SNP density. We then compared the average and distribution of all selection statistics across individual SNPs or windows located within bundle 0 and bundle 1a (labelled as ‘AMY region’) and located within chromosome 2: 135–138 Mb (labelled as the ‘*LCT* region’) with that of the rest of the genome using geom_stats() and geom_density() functions in ggplot2 (Supplementary Table 5 and Supplementary Figs. 6–18). We also used an outlier approach and focused on the top 0.05% of the test statistic across all windows genome-wide for modern populations of known subsistence, and considered estimates above this threshold to be strong signals of selection^80^. To improve detection power, we computed Fisher’s exact score^86^ from SNP ranks for the two selection statistics that were better able to identify signatures of selection at the AMY locus. Then, we investigated whether the scores computed from these statistics for SNPs located at the AMY locus were among the top 1% of Fisher’s exact scores estimated genome-wide (Supplementary Table 5 and Supplementary Fig. 18).

### Inference of recent positive selection in West Eurasian populations using ancient genomes

To determine whether changes in the frequency of different structural haplotypes over the past 12,000 years were consistent with positive selection, we first grouped amylase structural haplotypes (*n* = 11) into those with the ancestral number of amylase gene copies (three total) or with amylase gene duplications (five or more copies). We used three complementary approaches to infer the selection coefficient associated with duplication-containing haplotypes. First, we used ApproxWF^31^ to perform Bayesian inference of the selection coefficient from binned allele frequency trajectories. We ran ApproxWF for 101,0000 Markov chain Monte Carlo (MCMC) steps with parameters *n* = 10,000, *h* = 0.5 and pi = 1. We assumed a generation time of 30 years to convert the age of ancient samples from years to generations. The first 10,000 steps of the MCMC process were discarded in all analyses. Next, we used bmws (v0.1.0)^32^ to estimate the allele frequency trajectory and time-varying selection from genotype data with parameters -d diploid -l 4.5 -g 30 -n 10000 -t. We further ran 1,000 bootstrap replicates to obtain 95% credible intervals around our estimates. Last, we used an approximate Bayesian computation approach adapted and modified from ref. ^33^ to explicitly account for the demographic processes underlying the allele frequency changes. We performed extensive forward-in-time simulations using SLiM (v3.7.1)^87^ based on a well-established demographic model for West Eurasians^38^ that includes major population split and admixture events as well as population growth (Supplementary Table 11). We allowed three model parameters to vary across simulations: selection coefficient (*s*), the time of selection onset (*t*, in kyr bp) and the initial allele frequency in the ancestral population (*f*). Selection is only applied to known agricultural populations (that is, early farmers, Neolithic farmers, and Bronze Age to present-day Europeans), and its strength is assumed to be constant over time. These parameter values were set in evenly spaced intervals (that is, 21 values of *s* ∈ [−0.01, 0.04], 21 values of *t* ∈ [3, 15], 31 values of *f* ∈ [0.05, 0.8]), and 1,000 replicate simulations were run for each unique parameter combination. This resulted in 13,671,000 simulations in total. For each simulation, we calculated the difference between the observed and the expected binned allele frequency trajectories, accounting for uneven sampling in time and genetic ancestry. We then selected the top 0.1% of simulations (that is, 13,671 simulations) that best resemble the observed data to approximate the posterior distribution of model parameters. We also examined the allele frequency changes (that is, the difference between allele frequencies in the first and last time bin) across all neutral simulations with *s* = 0 and compared them with the observed allele frequency change in the data (Supplementary Fig. 25).

### Reporting summary

Further information on research design is available in the Nature Portfolio Reporting Summary linked to this article.

## Online content

Any methods, additional references, Nature Portfolio reporting summaries, source data, extended data, supplementary information, acknowledgements, peer review information; details of author contributions and competing interests; and statements of data and code availability are available at 10.1038/s41586-024-07911-1.

## Supplementary information

Supplementary Figures This Supplementary Figures file contains Supplementary Figures S1–S26.

Reporting Summary

Supplementary Tables This Supplementary Tables file contains Supplementary Tables S1–S11.

## Data Availability

All data used in this project are publicly available and described in the ‘Datasets’ section of the Methods. Copy number genotypes, structural haplotypes, haplotype deconvolutions and pangenome graphs can be found in the Supplementary tables and a GitHub repository (https://github.com/sudmantlab/amylase_diversity_project) that is archived in Zenodo (https://zenodo.org/doi/10.5281/zenodo.10995434)^88^. The HPRC data can be obtained at https://humanpangenome.org/data/. The 1KG data and the HGDP data can be obtained at https://www.internationalgenome.org/data/. The SGDP data can be obtained at https://www.simonsfoundation.org/simons-genome-diversity-project/. The joint 1KG and HGDP variant call set can be obtained at https://gnomad.broadinstitute.org/downloads#v3-hgdp-1kg. The ancient data are available on the European Nucleotide Archive under the accession codes PRJEB64656 and PRJEB50857. The raw GTEx expression data can be obtained at https://gtexportal.org/home/datasets. GTEx genetic data are available under restricted access at https://gtexportal.org/home/protectedDataAccess.

## Code availability

The code for haplotype deconvolution can be found in the following GitHub repository (https://github.com/raveancic/graph_genotyper) and is archived in Zenodo (https://zenodo.org/doi/10.5281/zenodo.10843493)^89^. All other code used in the paper can be found in the following GitHub repository (https://github.com/sudmantlab/amylase_diversity_project) and is archived in Zenodo (https://zenodo.org/doi/10.5281/zenodo.10995434)^88^.
