# Supplementary material for: Recurrent evolution and selection shape structural diversity at the amylase locus
Source: Nature. 2024 Sep 4;634(8034):617–25. doi: 10.1038/s41586-024-07911-1 (PMC11485256; doi:10.1038/s41586-024-07911-1)
Supplement: Supplementary file 1 — This Supplementary Figures file contains Supplementary Figures S1–S26. [file 41586_2024_7911_MOESM1_ESM.pdf]

nature portfolio

https://doi.org/10.1038/s41586-024-07911-1

Supplementary information

Recurrent evolution and selection shape structural diversity at the amylase locus

In the format provided by the authors and unedited

Supplementary Figures

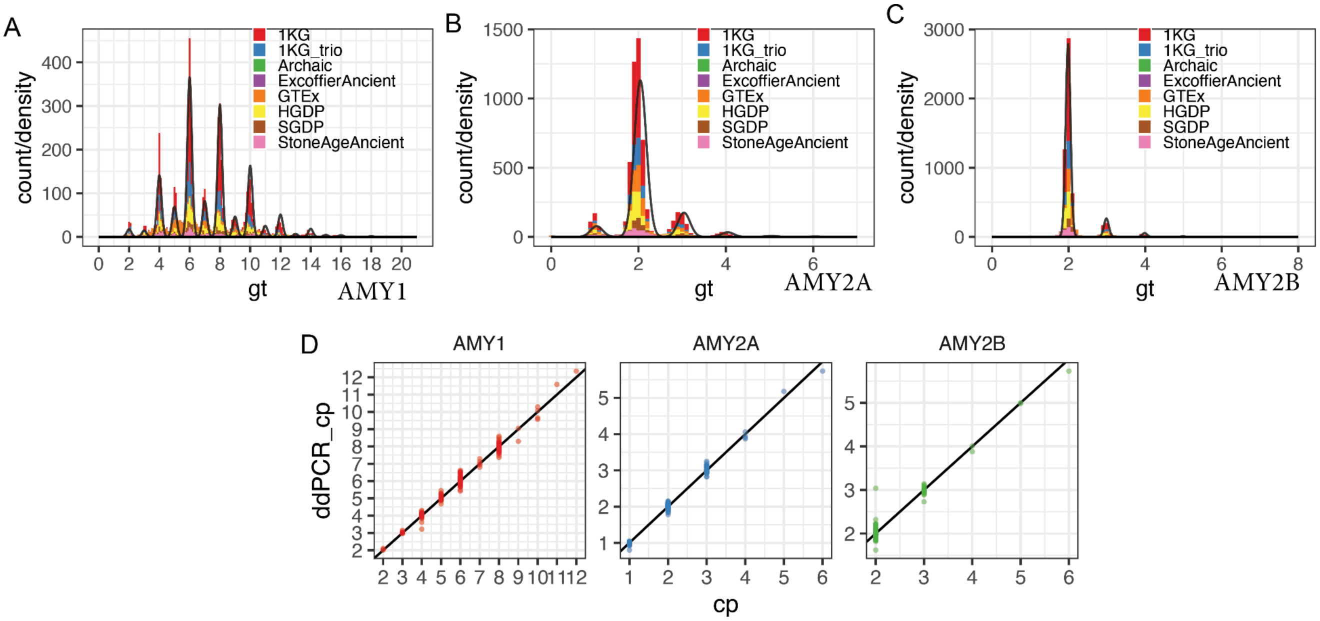

Figure S1 - Read depth-based genotyping. A-C) Histogram of “raw” read-depth based copy number across samples assessed in this study overlaid with a Gaussian Mixture Model (see methods) for *AMY1, AMY2A,* and *AMY2B*. **D)** Relationship between predicted copy number and digital droplet PCR across 149, 185, and 188 samples respectively with R^2 = 0.98, 0.99, and 0.96 respectively for *AMY1, AMY2A,* and *AMY2B.* Data from Usher et al, 2015.

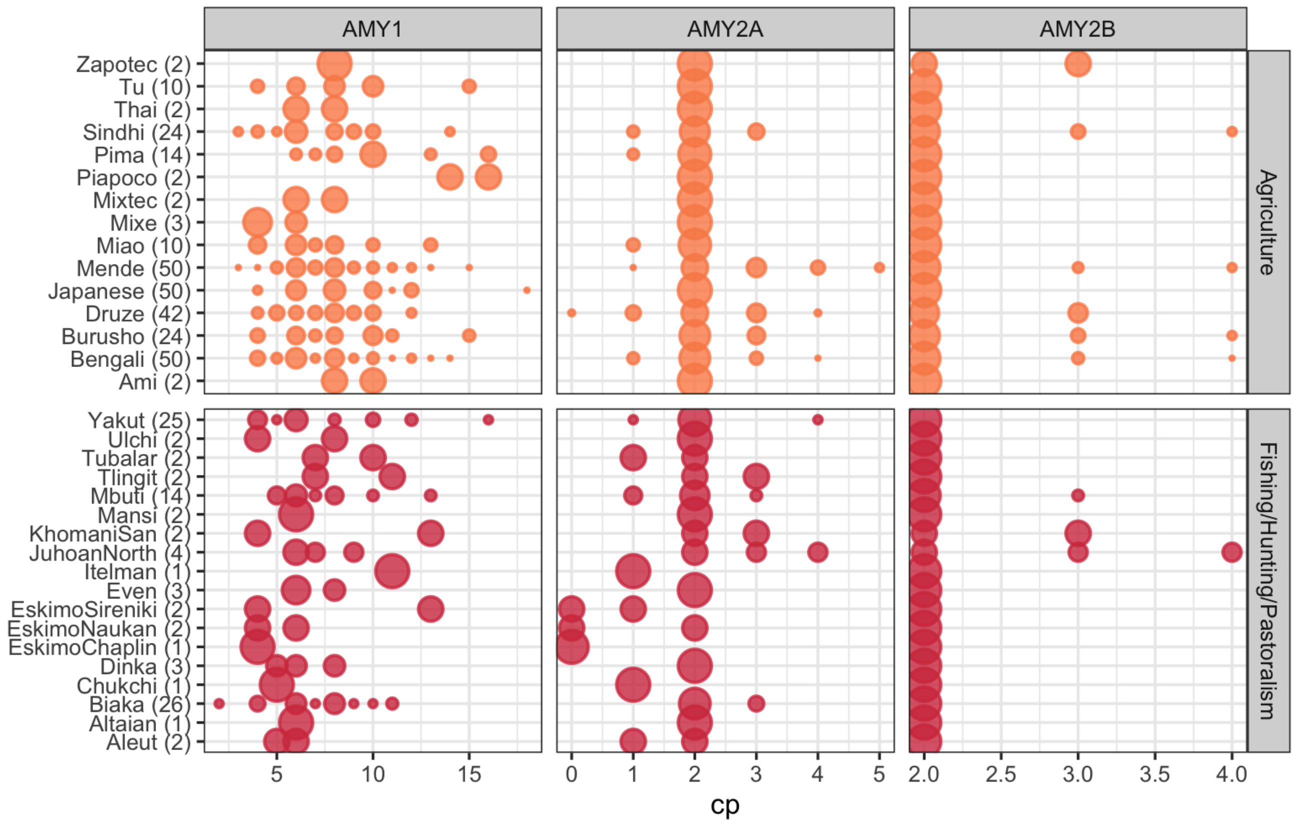

Fig S2 - Copy number by population for 33 populations with known traditional subsistence.

bundle 0 vs. bundle 1a

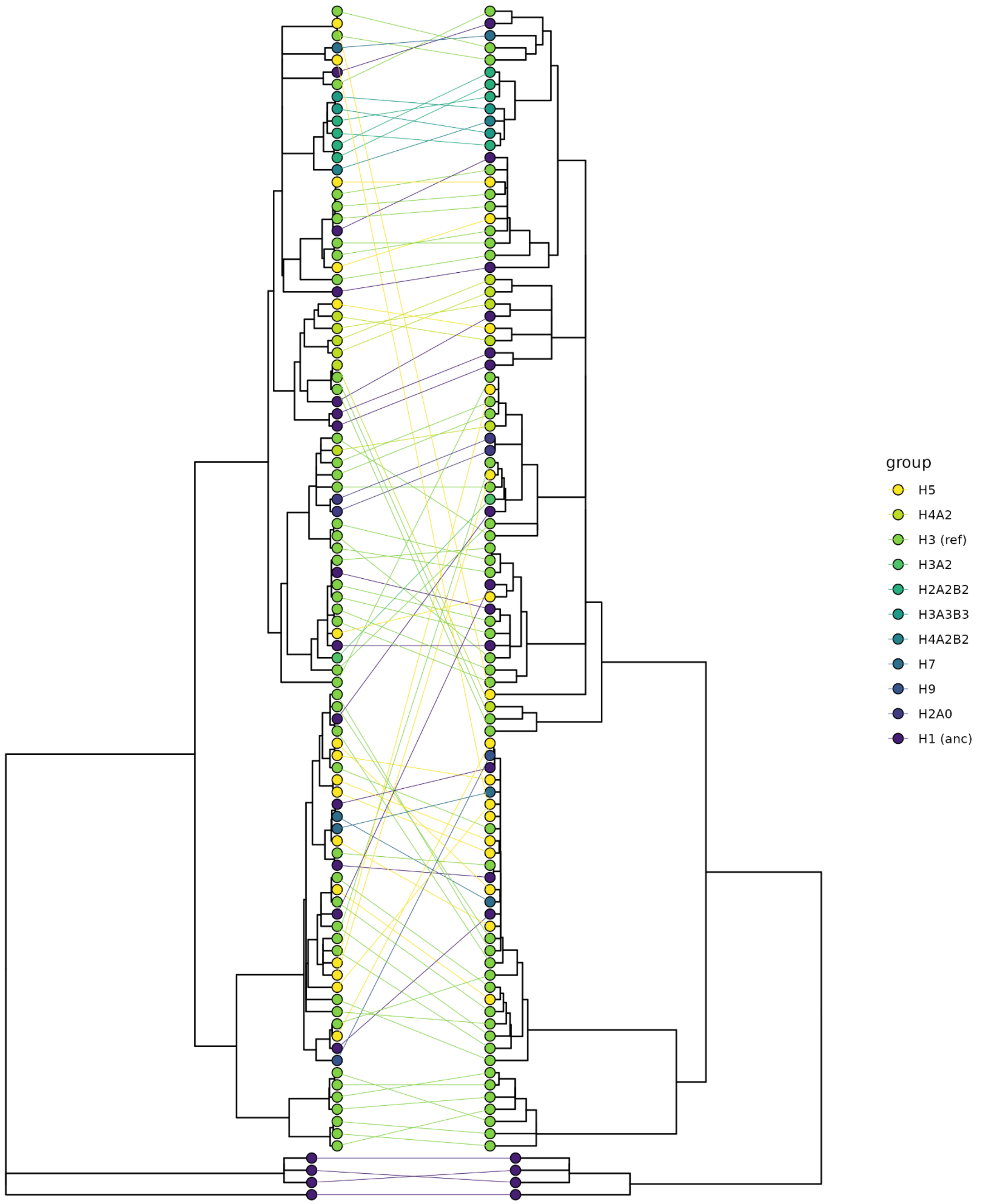

Figure S3 - Concordance between the coalescent trees based on the distal and proximal sequences flanking the amylase SVR (i.e. bundle 0 and bundle 1a). Tips are colored by their consensus haplotype structures (see **Fig 3C and Extended Data Fig 4A**), and sequences on the same haplotype background are connected by straight lines. The two trees are largely concordant, and the differences between them are likely due to the limited resolution offered by these short sequences, or rare recombination events. We choose to use bundle 0 in the main text because it is a longer sequence and offers higher resolution than bundle 1a.

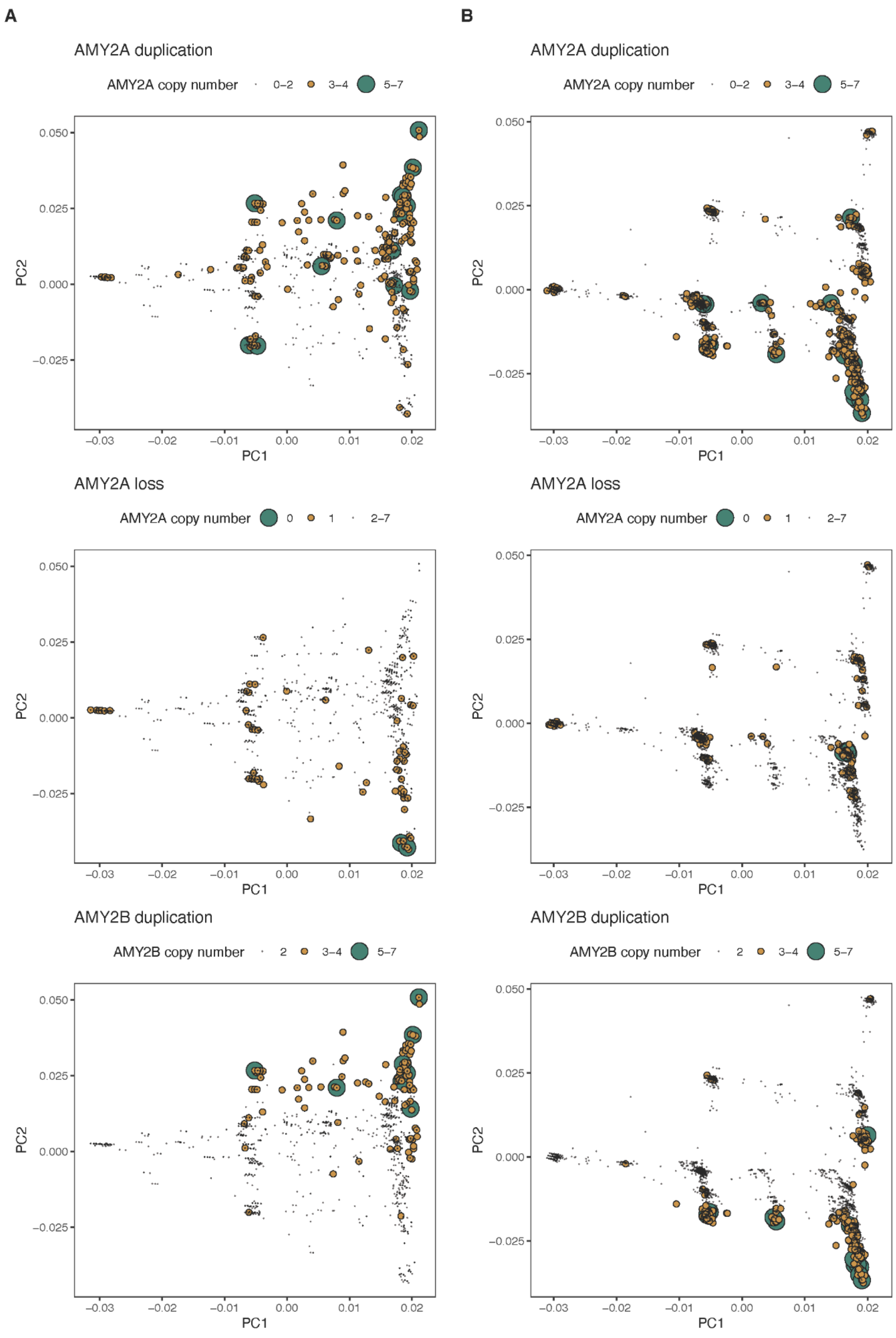

Figure S4 - PCAs highlighting the duplications and losses of AMY2A and the duplications of AMY2B in 3,395 diverse human genomes. PCAs were conducted using the **A)** distal (i.e. bundle 0) and **B)** proximal (i.e. bundle 1a) flanking region to the amylase SVR. All assembled haplotypes are hidden in these figures. They are exactly the same results as shown in the right column of Extended Data Fig 4C and Extended Data Fig 6, and the only difference is how amylase gene copy numbers are annotated.

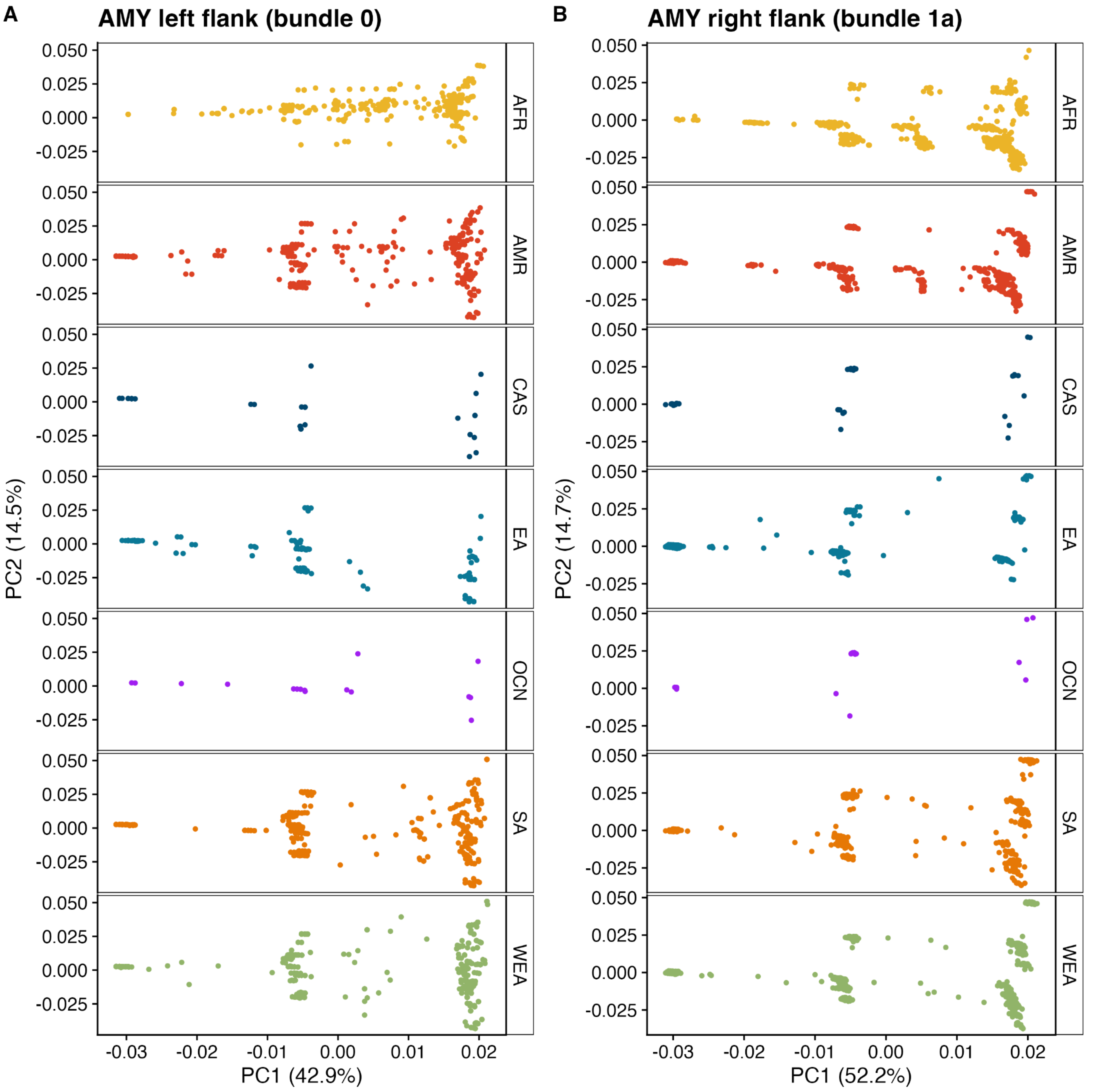

Figure S5 - PCAs for non-duplicated regions adjacent to the SVR according to different continental regions. These PCAs include 3,395 diverse diploid human genomes from using the **A)** distal (bundle 0) and **B)** proximal (bundle 1a) regions.

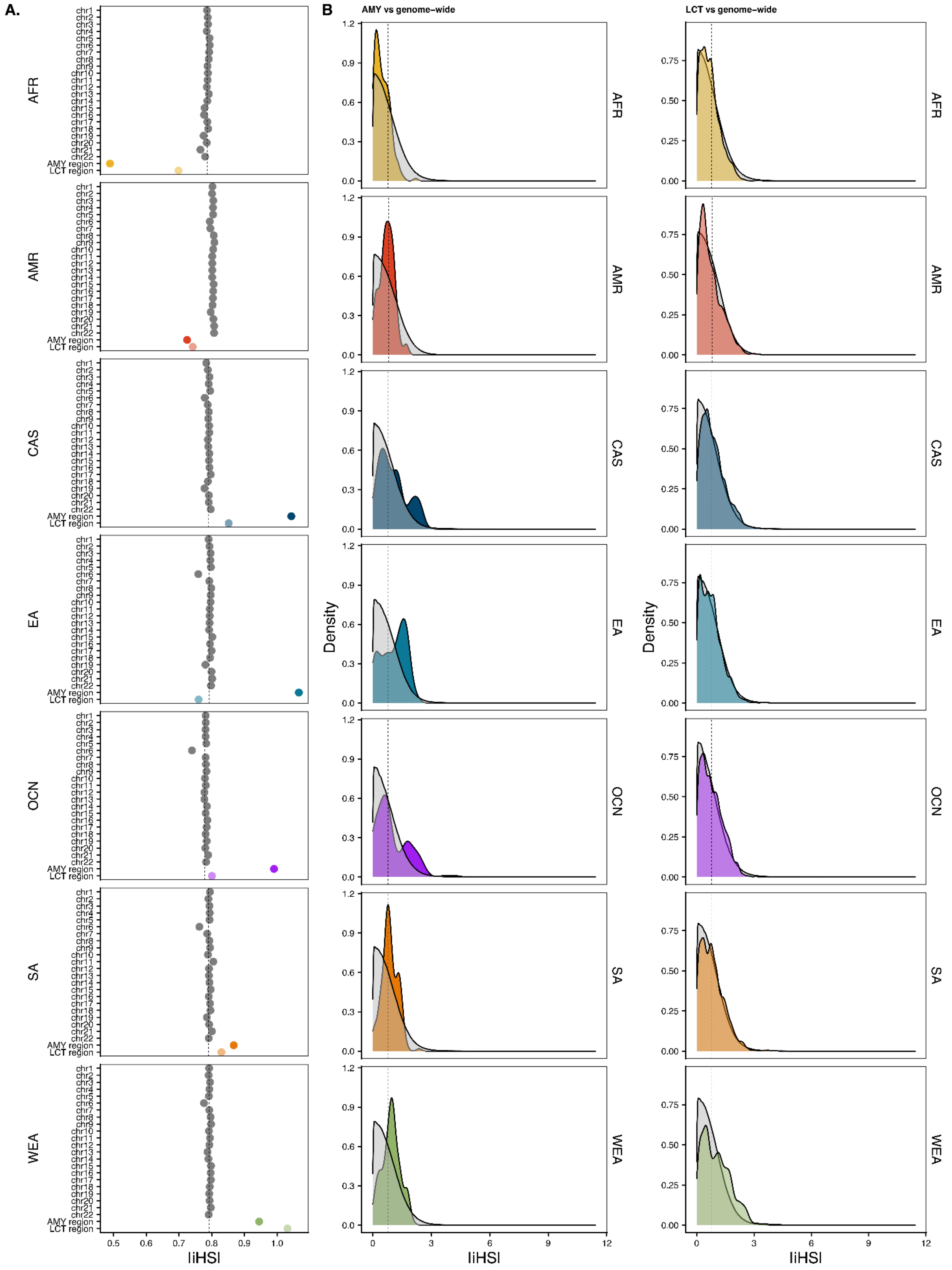

Figure S6 - iHS scan across SNPs for major continental regions. A) Mean absolute scores of individual SNPs along 22 autosomes, regions flanking amylase (b0 and b1a) and LCT region (chr2:135Mb-138Mb GRCh38). **B)** Distribution of absolute scores across AMY and LCT loci (colored by continental region) compared to genome-wide distribution (gray).

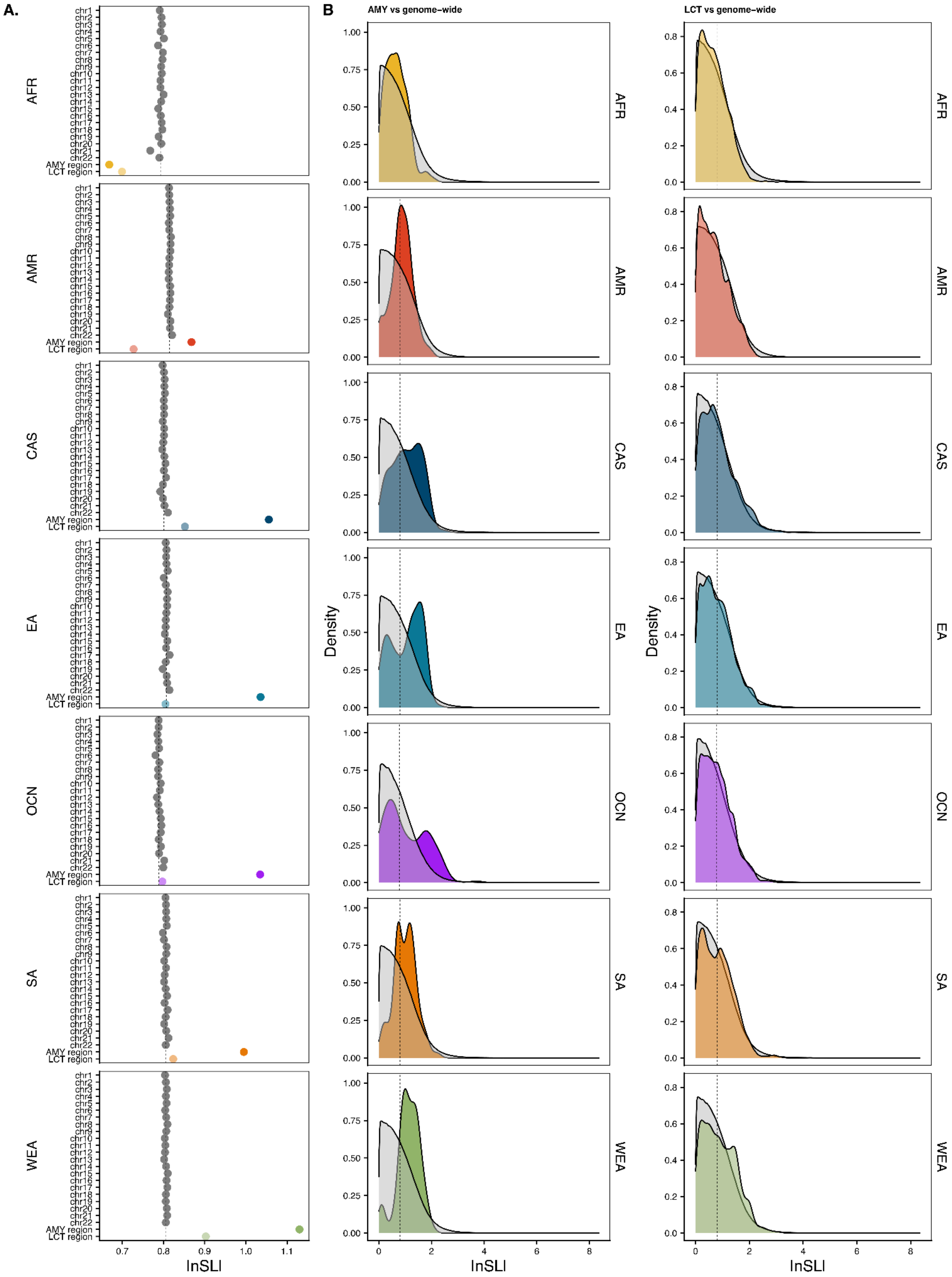

Figure S7 - nSL scan across SNPs for major continental regions. A) Mean absolute scores of individual SNPs along 22 autosomes, regions flanking amylase (b0 and b1a) and LCT region (chr2:135Mb-138Mb GRCh38). **B)** Distribution of absolute scores across AMY and LCT loci (colored by continental region) compared to genome-wide distribution (gray).

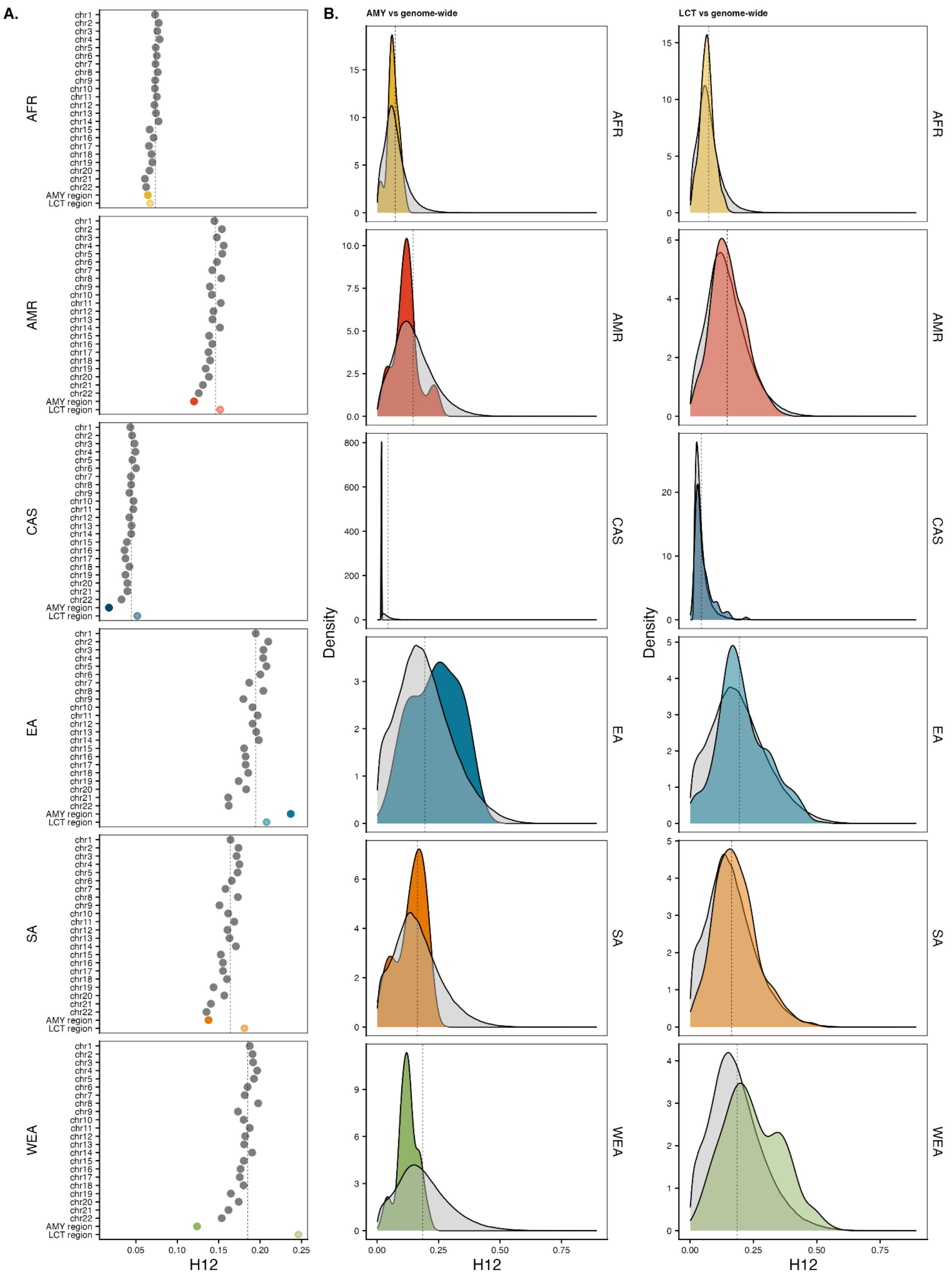

Figure S8 - H12 statistic across windows of 201 SNPs for major continental regions. A) Mean H12 along 22 autosomes, regions flanking amylase (b0 and b1a) and LCT region (chr2:135Mb-138Mb GRCh38). **B)** Distribution of H12 across AMY and LCT loci (colored by continental region) compared to genome-wide distribution (gray). Populations lacking 201 SNPs windows for AMY were removed.

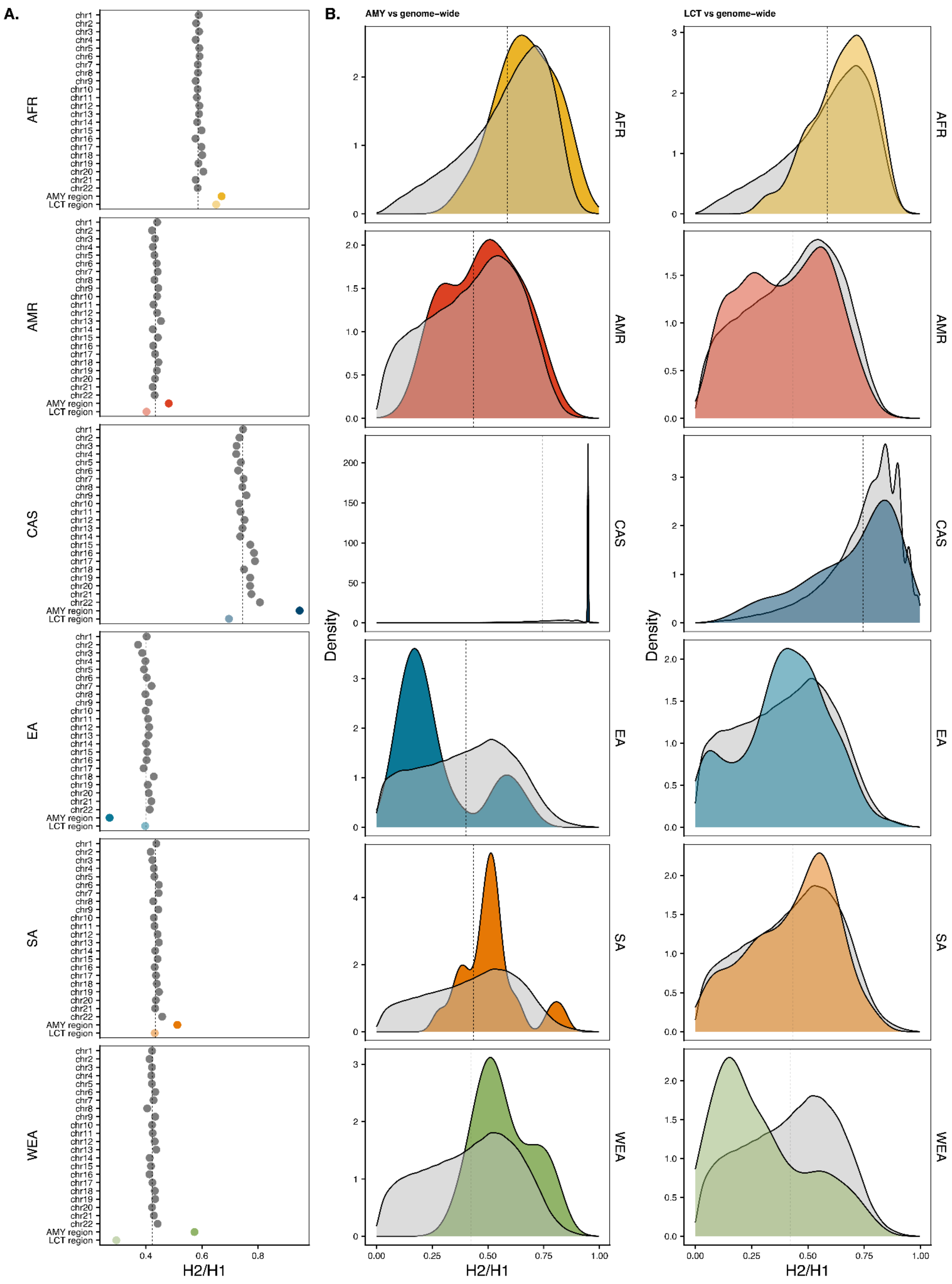

Figure S9 - H2/H1 across windows of 201 SNPs for major continental regions. A) Mean H2/H1 along 22 autosomes, regions flanking amylase (b0 and b1a) and LCT region (chr2:135Mb-138Mb GRCh38). **B)** Distribution of H2/H1 across AMY and LCT loci (colored by continental region) compared to genome-wide distribution (gray). Populations lacking 201 SNPs windows for AMY were removed.

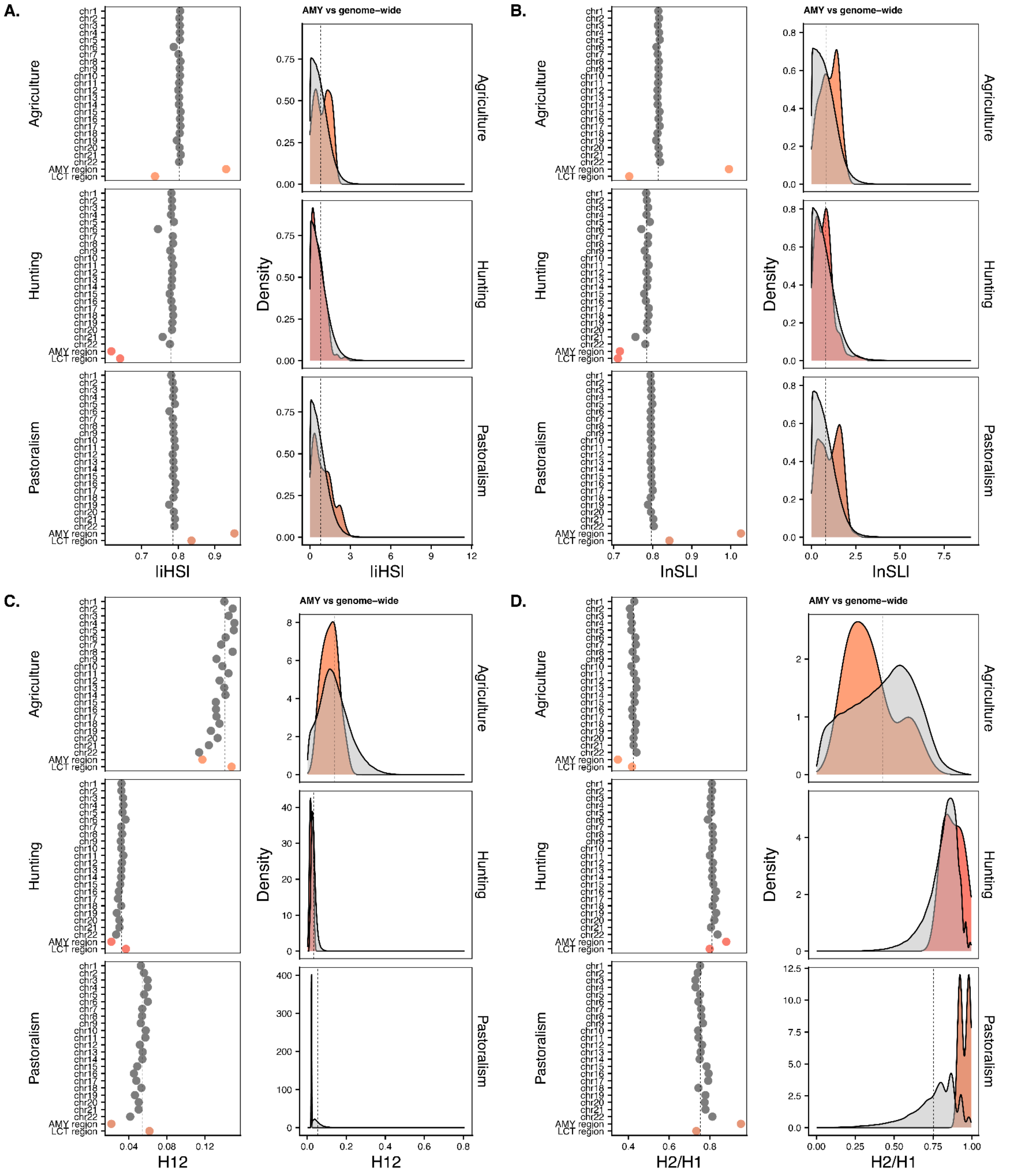

Figure S10 - iHS (A), nSL (B), H12 (C), and H2/H1 (D) for major subsistence groups. Mean scores for individual SNPs along 22 autosomes and across regions flanking amylase (b0 and b1a), beside the distribution of scores across AMY loci (colored by subsistence) compared to genome-wide distribution (gray).

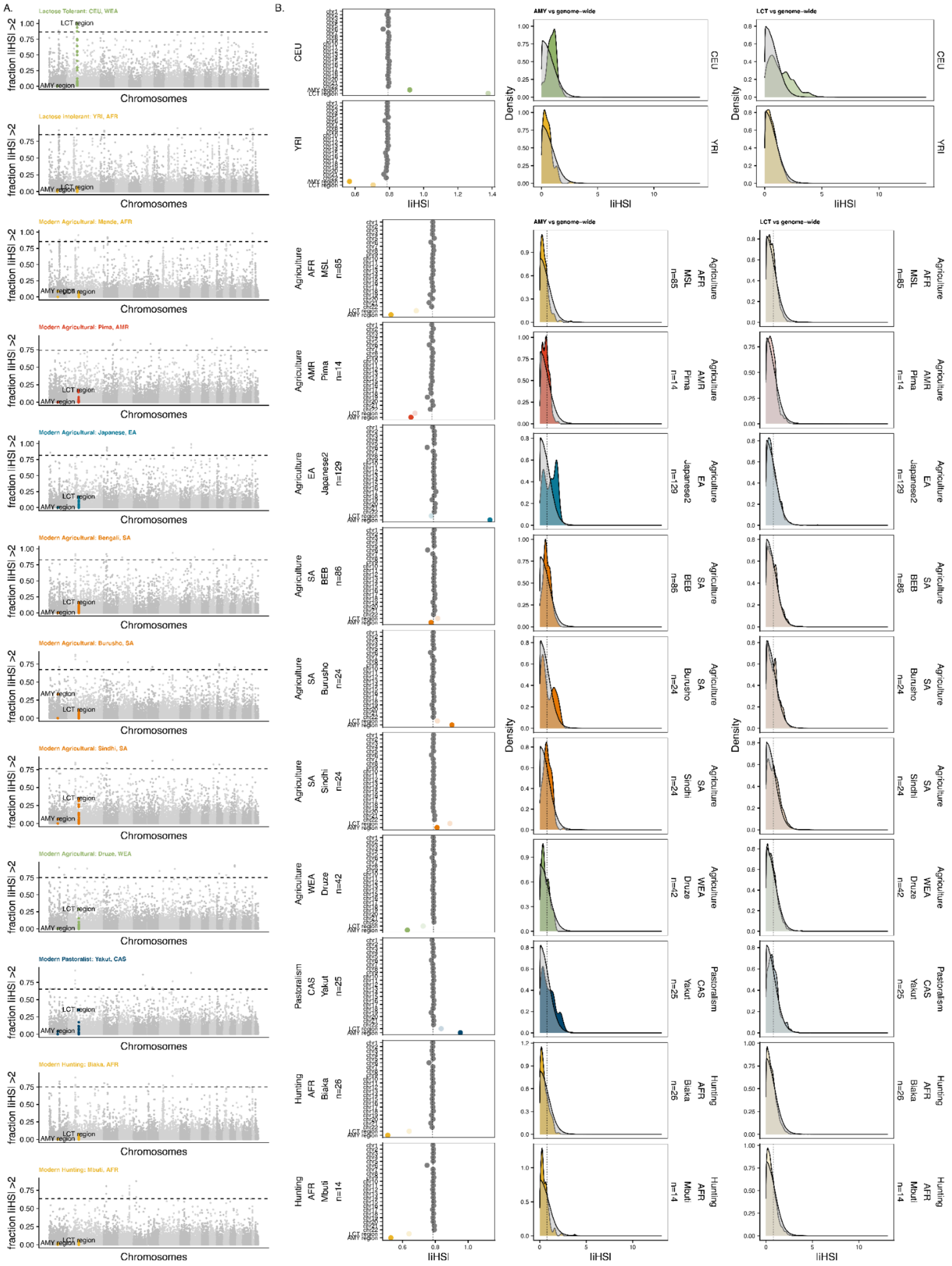

Figure S11 - iHS scan for modern populations of known subsistence. A) Manhattan plot of the proportion of SNPs in 100 Kb windows with absolute scores > 2; dashed line threshold separates regions above the 99.95th percentile. **B)** Mean absolute scores for individual SNPs along 22 autosomes, regions flanking amylase (b0 and b1a) and LCT region (chr2:135Mb-138Mb GRCh38) beside the distribution across AMY and LCT loci compared to genome-wide distribution.

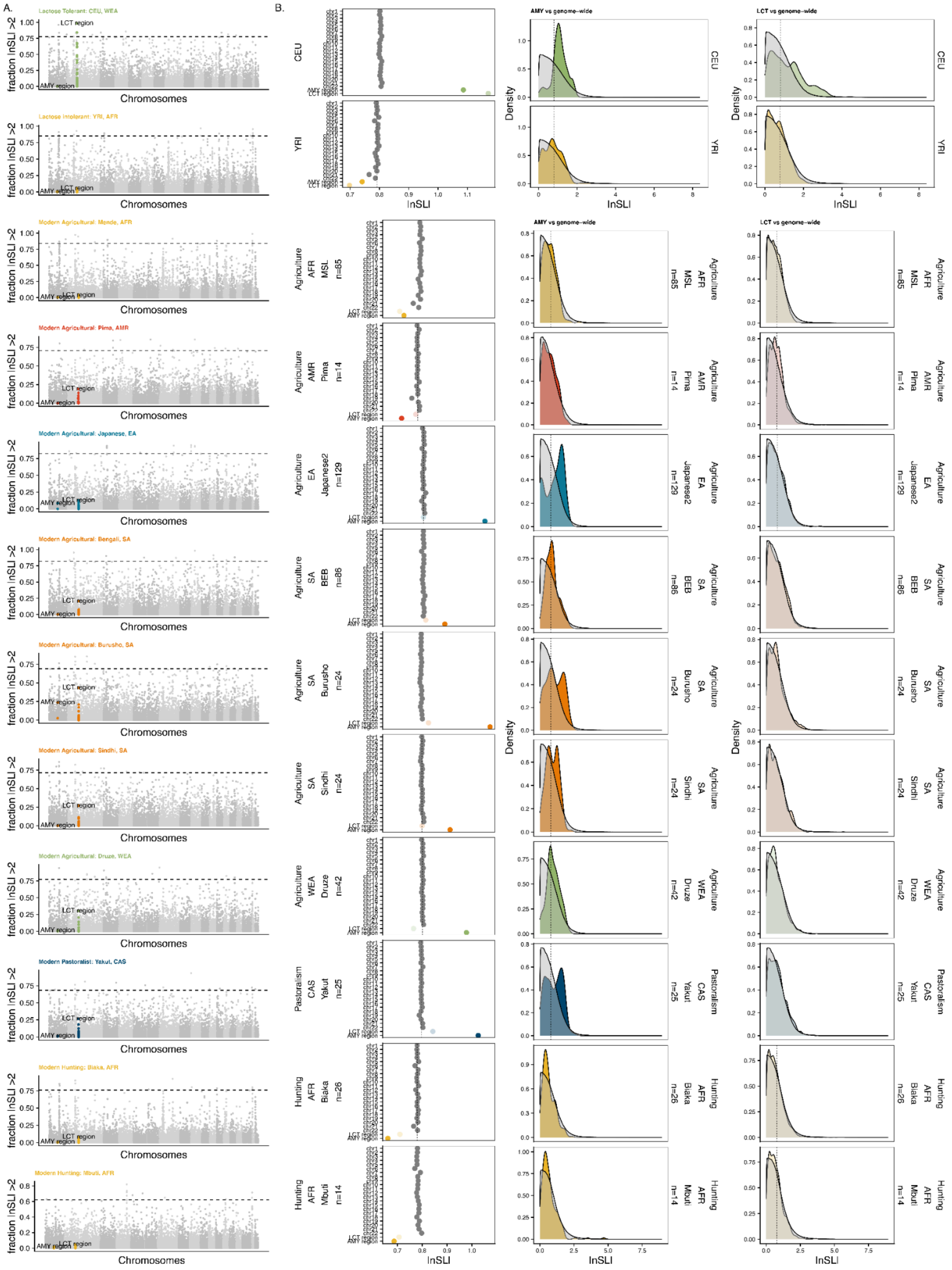

Figure S12 - nSL scan for modern populations of known subsistence. A) Manhattan plot of the proportion of SNPs in 100 Kb windows with absolute scores > 2; dashed line threshold separates regions above the 99.95th percentile. **B)** Mean absolute scores for individual SNPs along 22 autosomes, regions flanking amylase (b0 and b1a) and LCT region (chr2:135Mb-138Mb GRCh38) beside the distribution across AMY and LCT loci compared to genome-wide distribution.

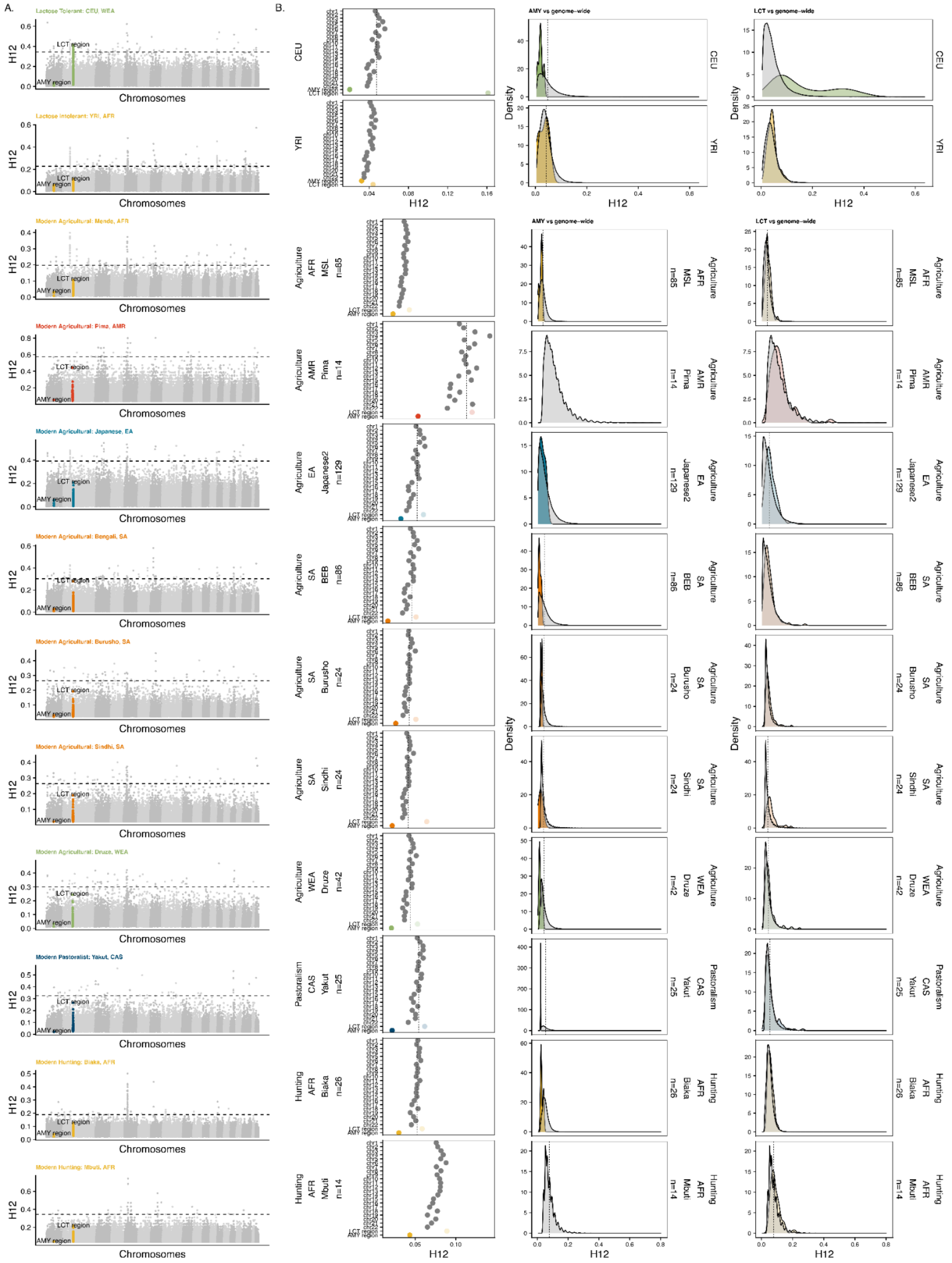

Figure S13 - H12 for modern populations of known subsistence. A) Manhattan plot of the statistic calculated on 201 SNP windows with 100 SNP overlap; dashed line threshold separates regions above the 99.95th percentile. **B)** Mean H12 for individual windows along 22 autosomes, regions flanking amylase (b0 and b1a) and LCT region (chr2:135Mb-138Mb GRCh38) beside the distribution across AMY and LCT loci compared to genome-wide distribution.

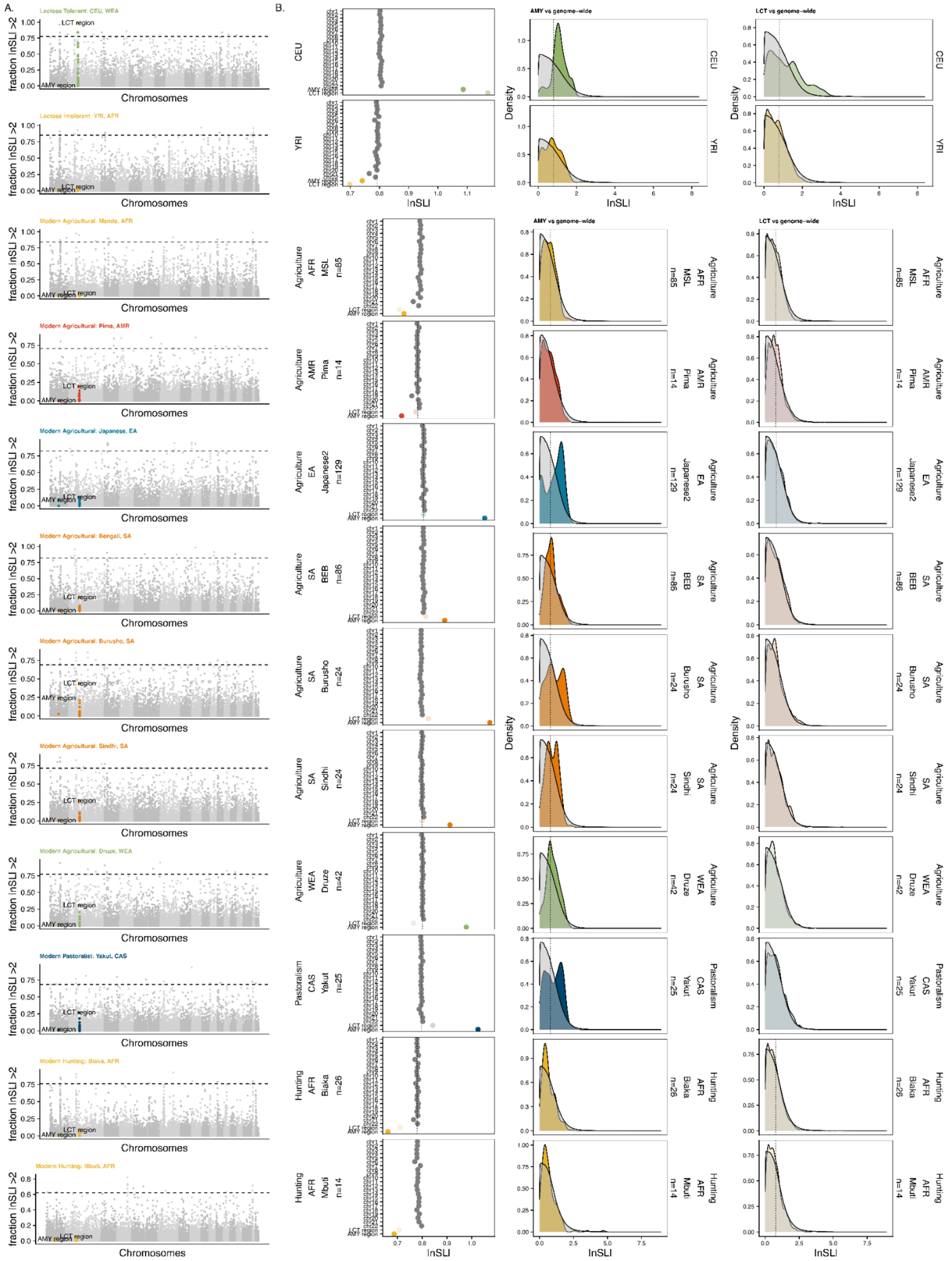

Figure S14 - H2/H1 for modern populations of known subsistence. A) Manhattan plot of the statistic calculated on 201 SNP windows with 100 SNP overlap; dashed line threshold separates regions above the 99.95th percentile. **B)** Mean H2/H1 for individual windows along 22 autosomes, regions flanking amylase (b0 and b1a) and LCT region (chr2:135Mb-138Mb GRCh38) beside the distribution across AMY and LCT loci compared to genome-wide distribution.

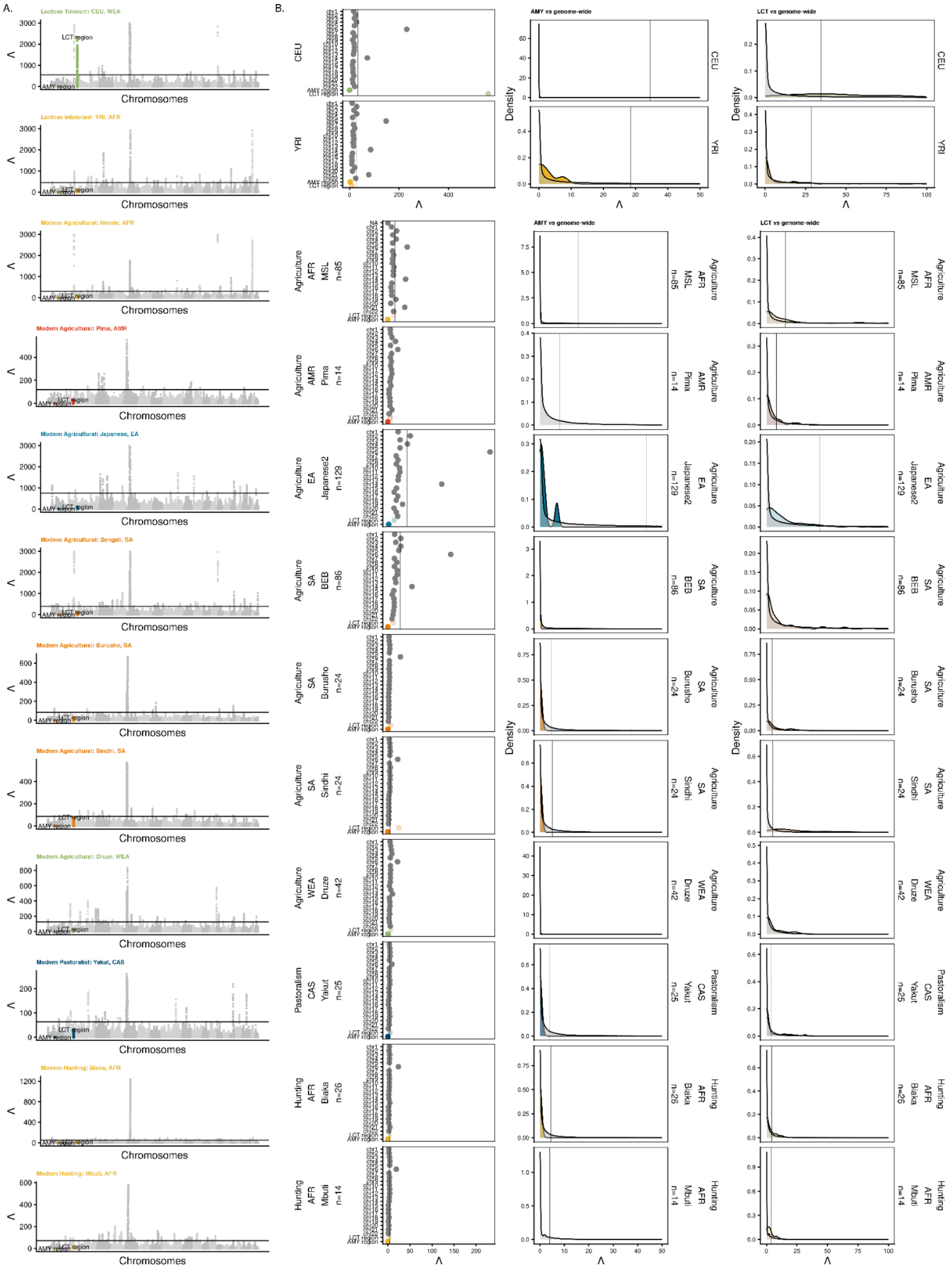

Figure S15 - Λ for modern populations of known subsistence. A) Manhattan plot of the *saltiLassi* statistic Λ calculated on 201 SNP windows with 100 SNP overlap along the genome; dashed line threshold separates regions above the 99.95th percentile. **B)** Mean Λ for individual windows along 22 autosomes, regions flanking amylase (b0 and b1a) and LCT region (chr2:135Mb-138Mb GRCh38) beside the distribution across AMY and LCT loci compared to genome-wide distribution.

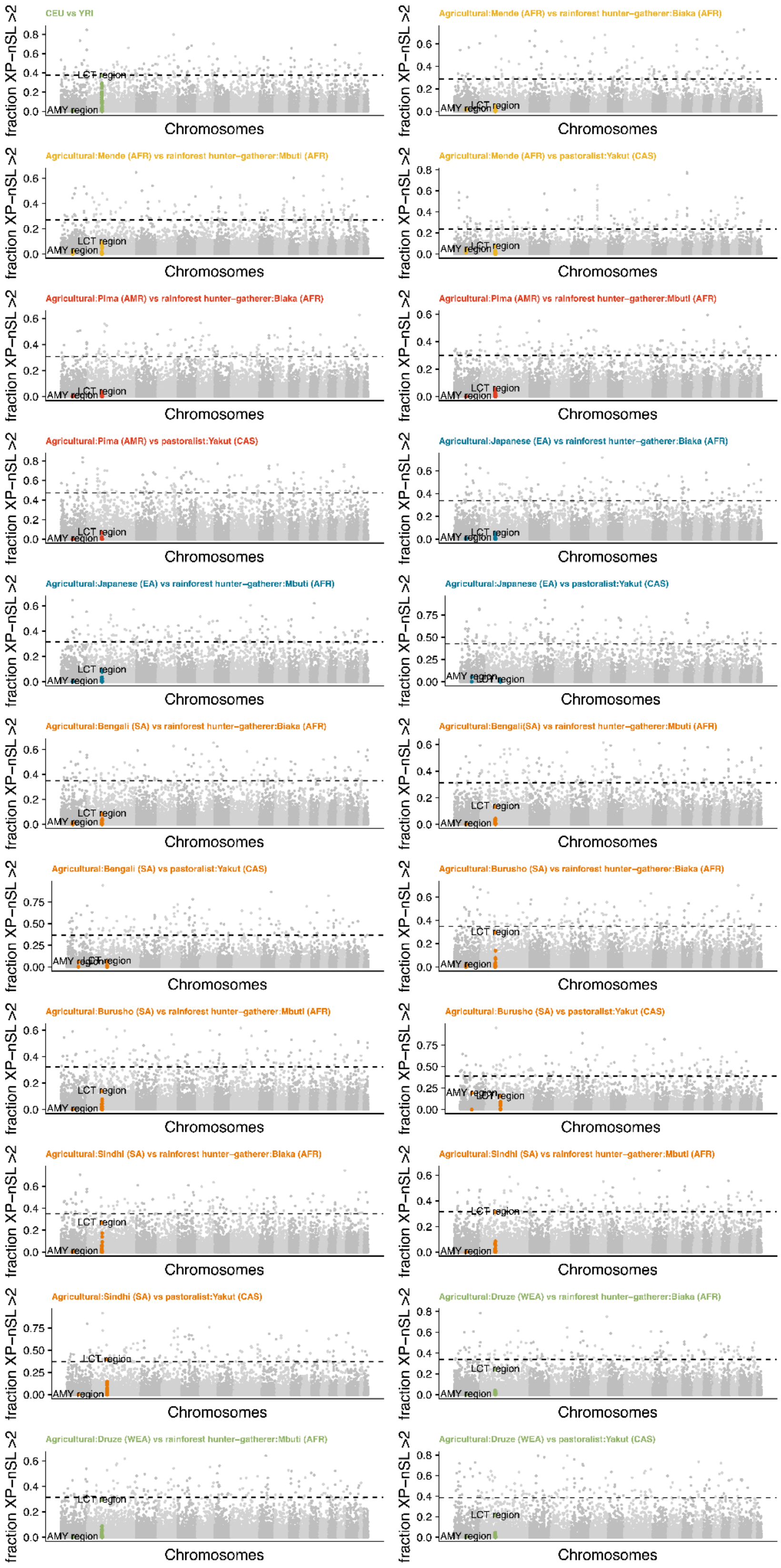

Figure S16 - XP-nSL between populations with different subsistence. Manhattan plot of the proportion of SNPs in 100 Kb windows throughout the genome with XP-nSL scores > 2 ; dashed line threshold separates regions above the 99.95th percentile.

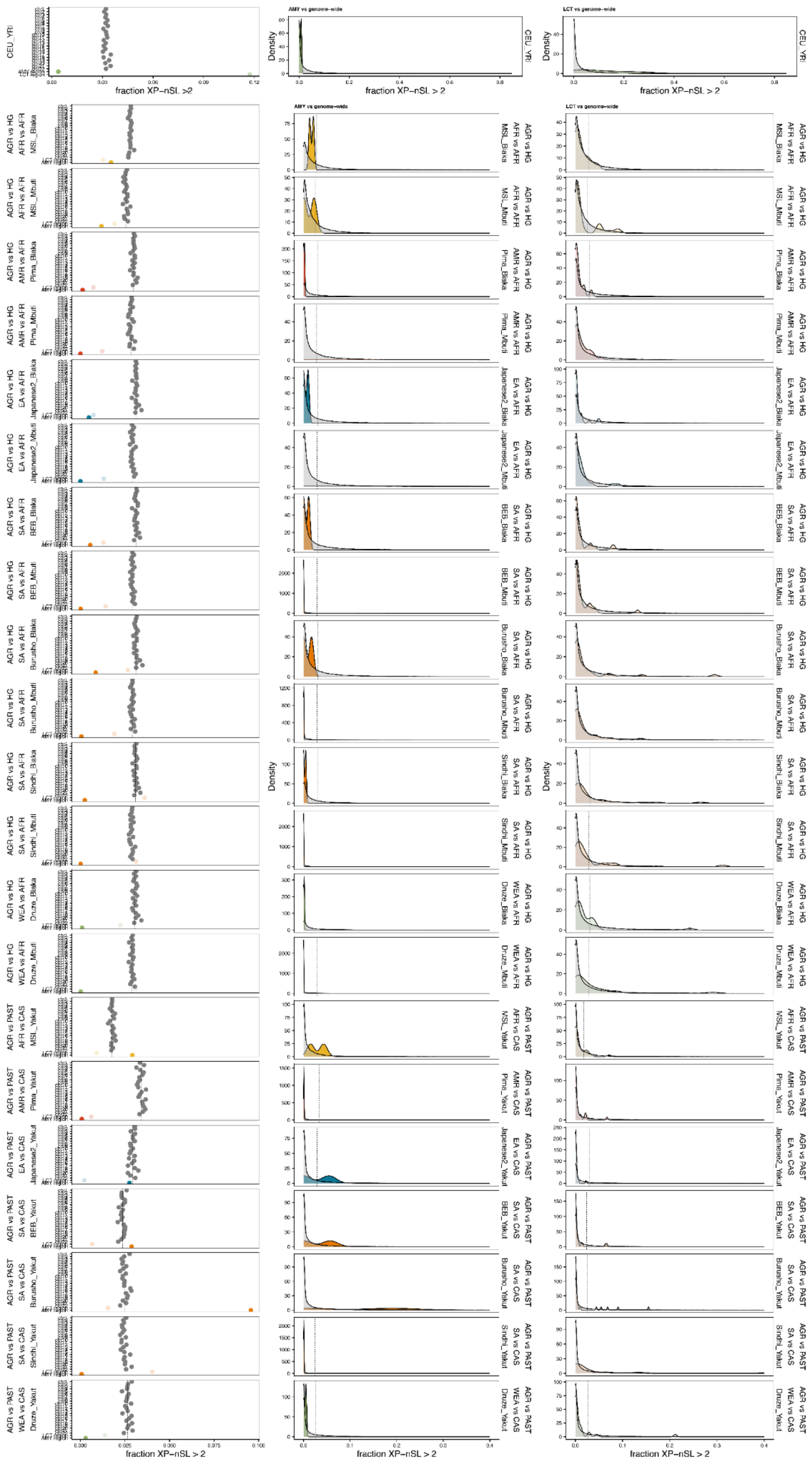

Figure S17 - XP-nSL between populations with different subsistence. Mean proportion of SNPs in 100 Kb windows with XP-nSL scores > 2 along 22 chromosomes compared to regions flanking amylase (b0 and b1a) and LCT region (chr2:135Mb-138Mb GRCh38), followed by the distribution across AMY and LCT loci compared to genome-wide distribution.

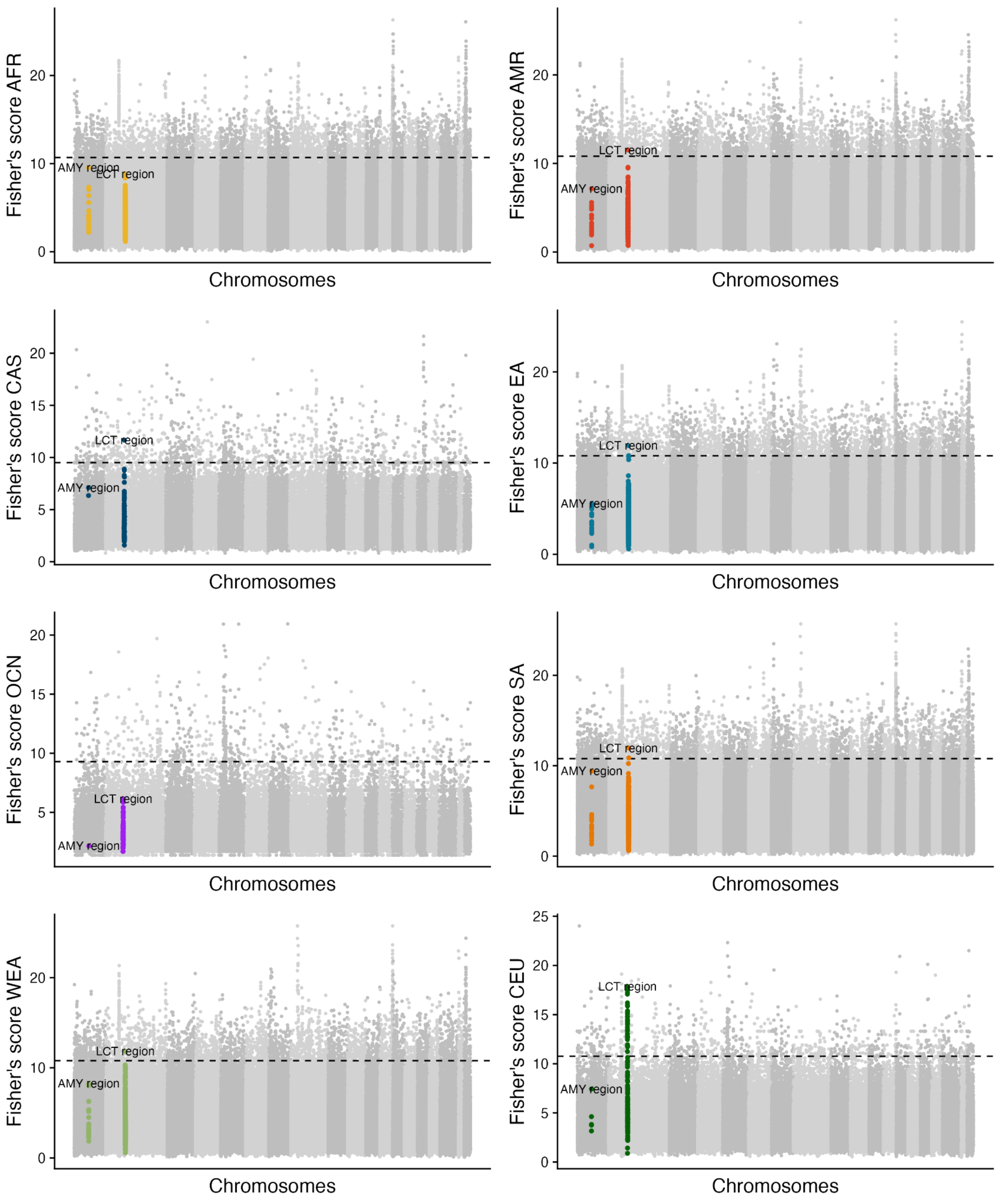

Figure S18 - Fisher's score for combined selection statistics for major continental regions and in CEU. Manhattan plots of Fisher's score, a metric computed from SNP ranks for H12 and H2/H1 statistics calculated across sliding windows of 201 SNPs; dashed line threshold separates regions above the 99th percentile of the genome-wide empirical distribution.

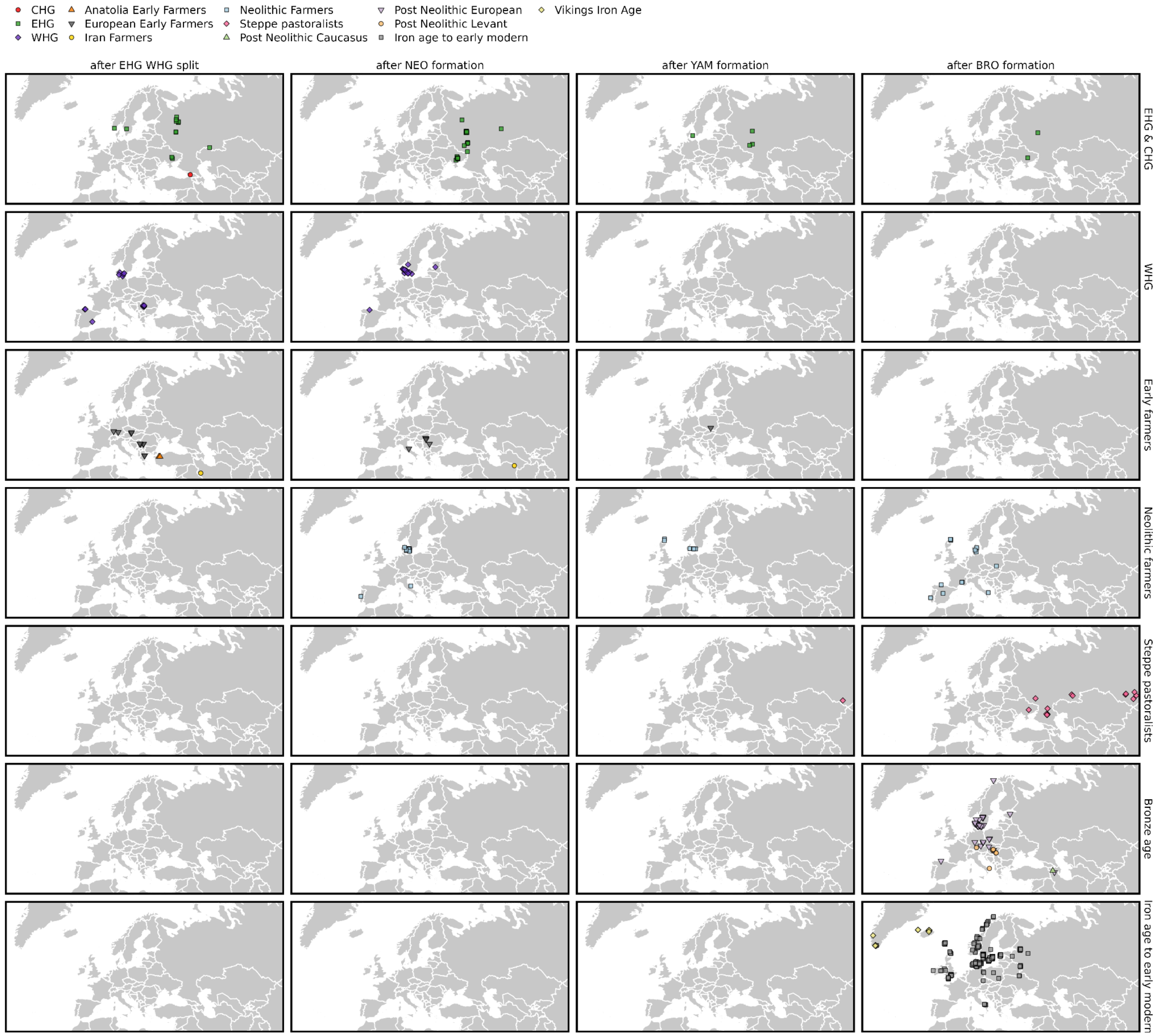

Figure S19. Map of 533 ancient West-Eurasian samples facetted by population and epoch. Epochs are assigned based on the timing of major population split and admixture events in a well-established demographic model of West Eurasians (NEO: Neolithic Farmer, YAM: Steppe pastoralist, BRO: Bronze age Europeans). The color and shape of points correspond to more precise population labels when applicable.

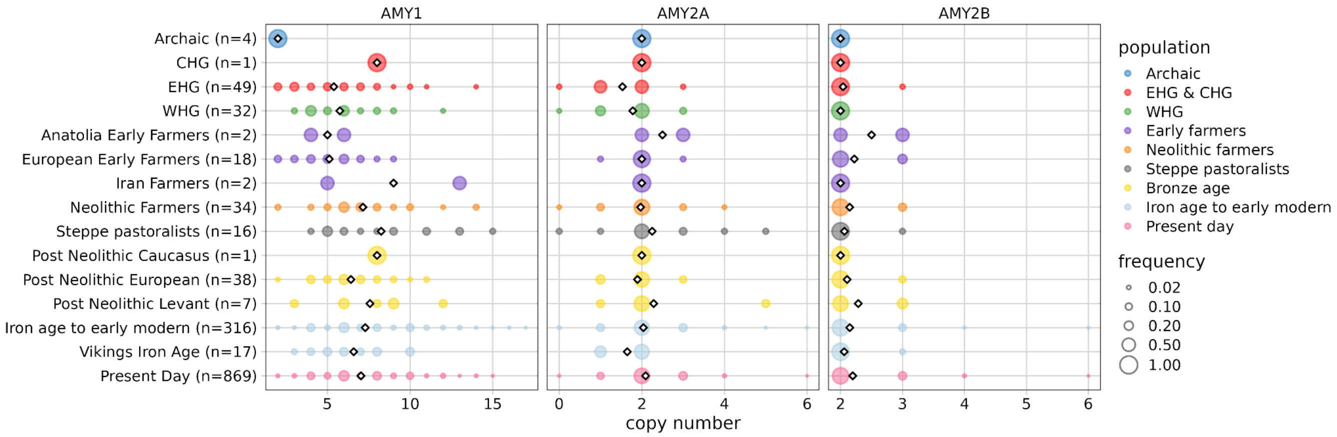

Figure S20. The distribution of *AMY1, AMY2A,* and *AMY2B* copy numbers in ancient and modern populations of West Eurasia. Broad population labels are divided into more precise ones when applicable, but points are still colored by the broad population labels. The size of each point is proportional to the proportion of individuals in the population with that genotype. Diamonds indicate the population mean.

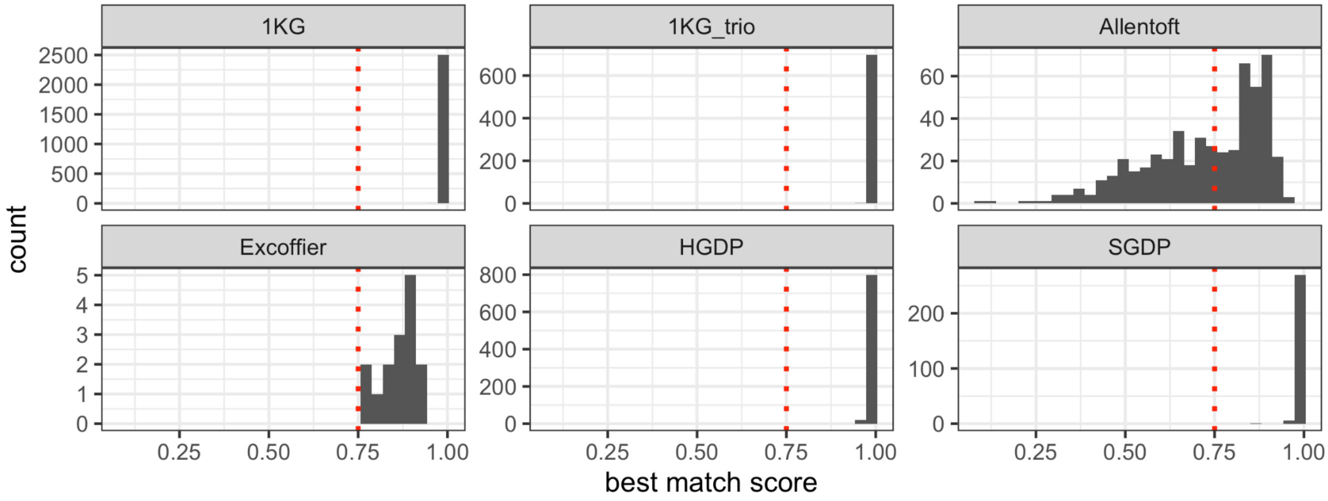

Figure S21 - Haplotype deconvolution with ancient genomes. Haplotype deconvolution scores (x-axis, cosine similarity score) for the best matching genotype for short-read sequenced individual. Scores tend to be lower for low-coverage ancient genomes thus individuals below a cutoff of 0.75 were discarded.

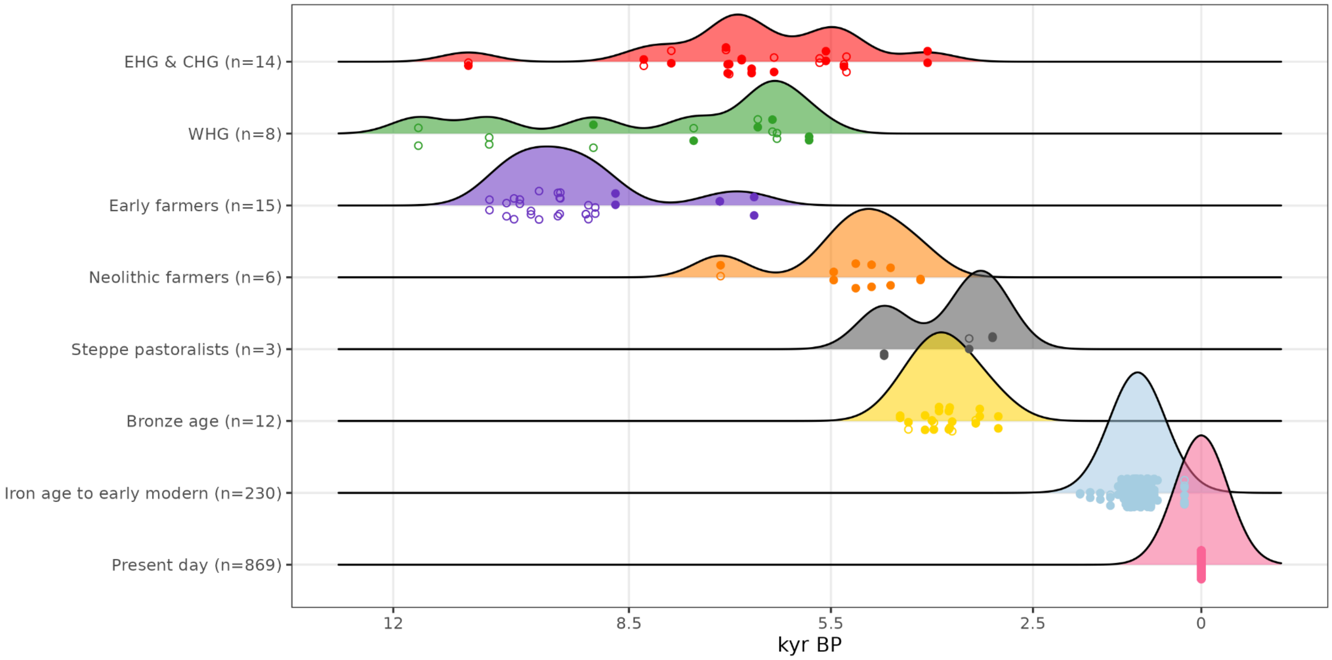

Figure S22. The age distribution of West Eurasian samples that had high-confidence haplotype assignment. Each sample is represented by two points corresponding to its two haplotypes. Filled circles represent duplication-containing haplotypes (with 5 or more amylase copies), and open circles represent the ancestral H1[a] and the H2A0 haplotypes (with 3 amylase copies each).

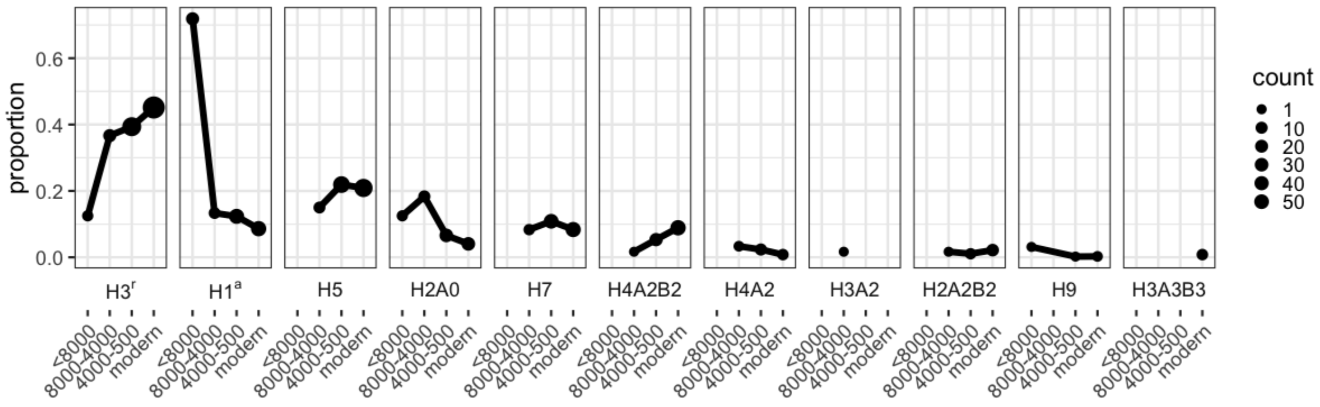

Figure S23 - Haplotype frequencies through time. Haplotype frequencies as a function of time ~4000 year bins in West Eurasian populations.

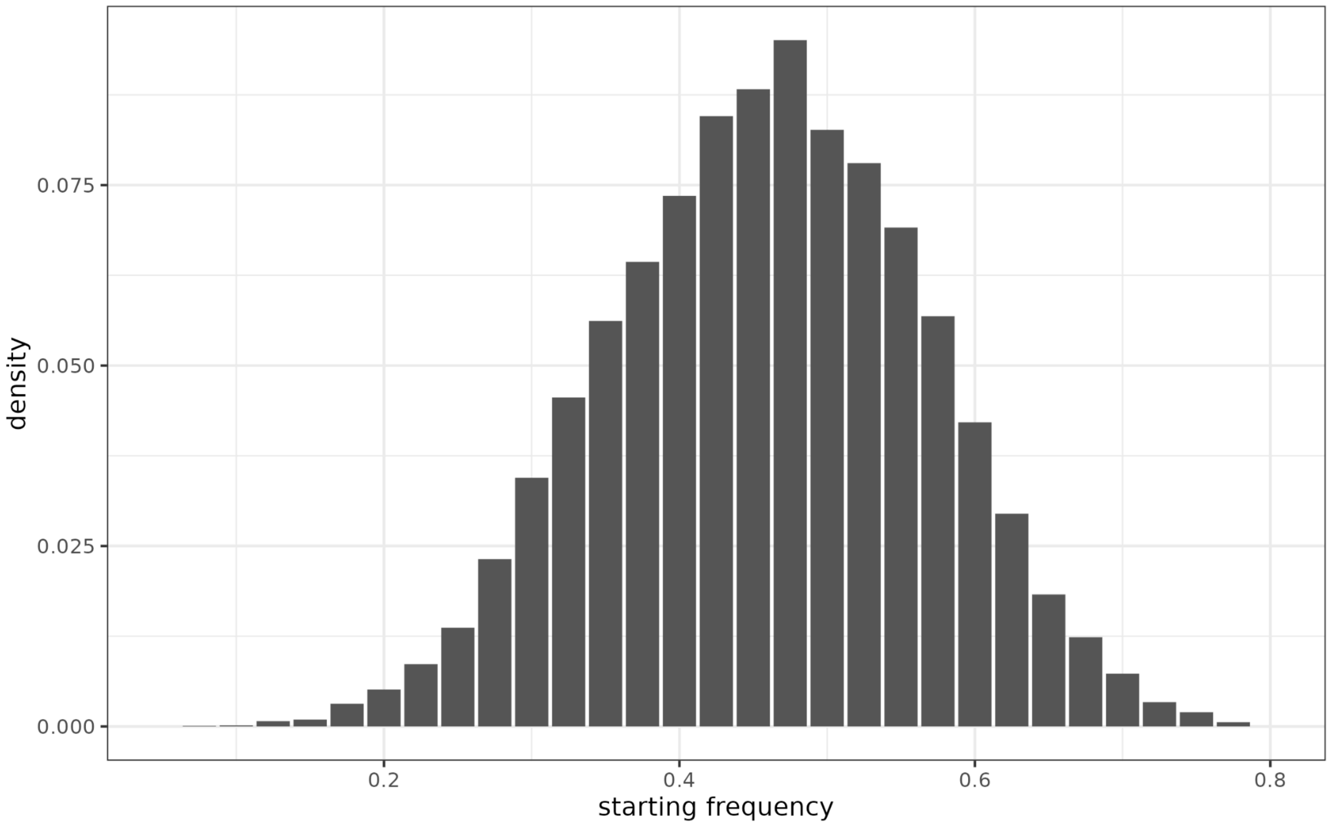

Figure S24. Posterior distribution of the frequency of duplication-containing haplotypes in the common ancestors of West Eurasian populations 45 kya BP inferred from the ABC analysis.

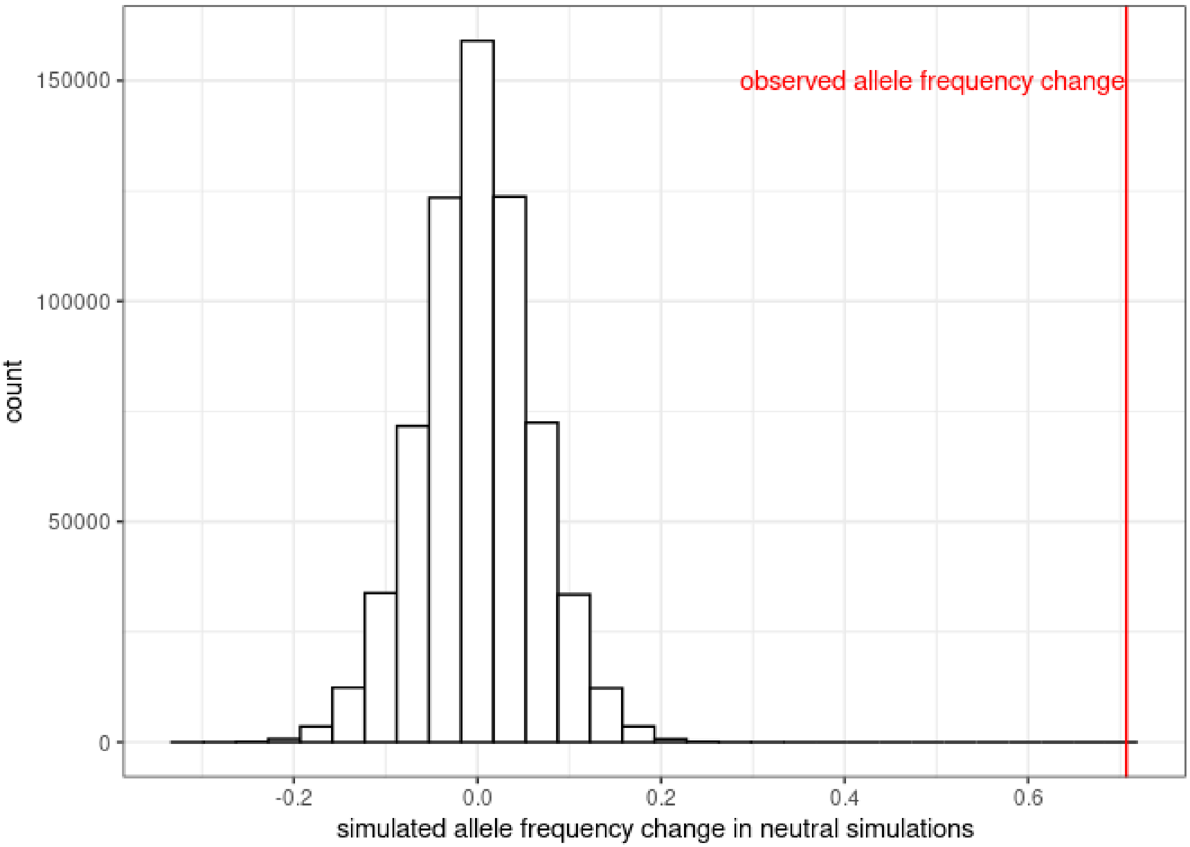

Figure S25. The distribution of allele frequency change (i.e. the difference between the allele frequencies of duplication-containing haplotypes in the first and last time bin) in all 651,000 neutral simulations (i.e. with s=0). None of these are as extreme as the observed allele frequency change in the data (shown in red), corresponding to a p-value<1.6×10^{-6}.

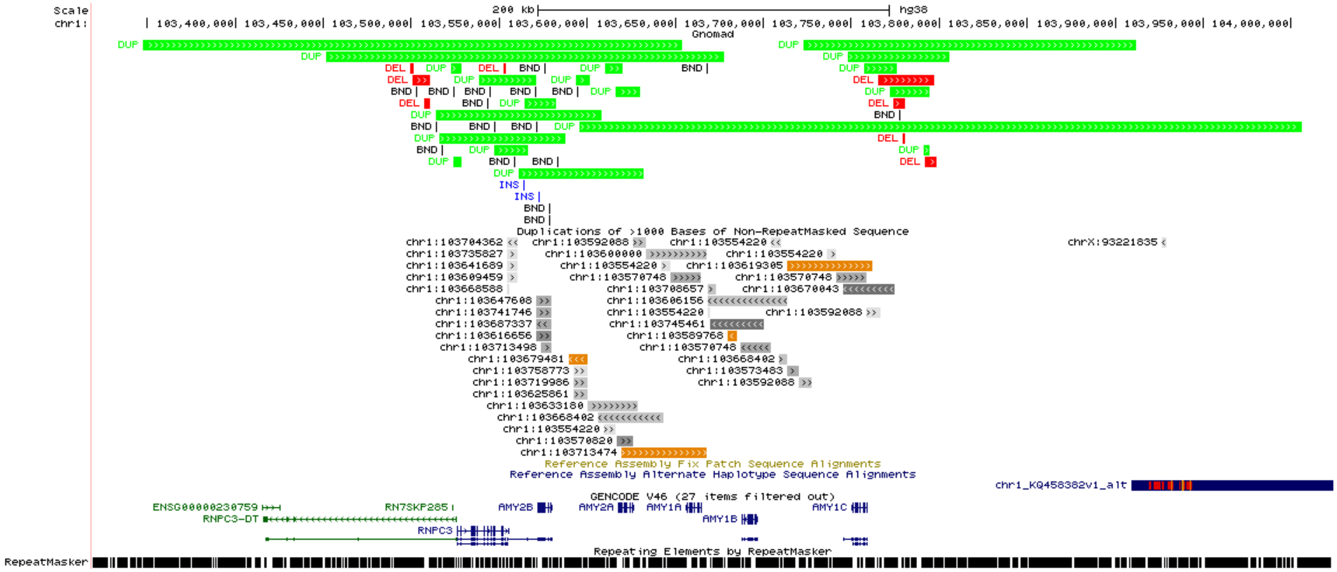

Figure S26. A browser image showing gnomAD SV calls from 14,891 individuals colored by SV type. Duplications [DUP] in green, deletions [DEL] in red, insertions [INS] in blue, and unresolved single end breakpoints [BND] in black. All SV calls between 1kb and 400kb (n=46) are shown. The segmental duplication blocks are shown below as well as gene coordinates. SVs are identified as projections against the human reference genome making it extremely challenging to gain insight into the different structural haplotypes. These calls vary widely in size and are nested within one another and do not correspond to the repeat units (i.e. principal bundles) identified using long read assemblies, nor to individual amylase gene duplications. This is because the complex structures of amylase (**Fig 2**) cannot be projected as simple SV calls onto the reference, hence the need for pangenome methods. An example of this is the ancestral haplotype H1 (i.e. the least complex structure, found at ~10% frequency in different continental populations), which ideally should be observed as a deletion call spanning two AMY1 genes. However, this dataset shows no evidence of such deletions or of this haplotype at all. In addition, no inversions were identified, despite the many inversions found at this locus from analyzing long read haplotype assemblies. Thus, despite the power of large short read datasets, their utility can be improved immensely by pangenome graphs based on long read assemblies (e.g. through haplotype deconvolution).
