# Supplementary material for: Recurrent evolution and selection shape structural diversity at the amylase locus
Source: Nature. 2024 Sep 4;634(8034):617–25. doi: 10.1038/s41586-024-07911-1 (PMC11485256; doi:10.1038/s41586-024-07911-1)
Supplement: Supplementary file 2 — Reporting Summary [file 41586_2024_7911_MOESM2_ESM.pdf]

nature portfolio

Corresponding author(s): Peter Sudmant

Last updated by author(s): 2024/07/15

Reporting Summary

Nature Portfolio wishes to improve the reproducibility of the work that we publish. This form provides structure for consistency and transparency in reporting. For further information on Nature Portfolio policies, see our Editorial Policies and the Editorial Policy Checklist.

Statistics

For all statistical analyses, confirm that the following items are present in the figure legend, table legend, main text, or Methods section.

n/a	Confirmed	
☐	☒	The exact sample size (*n*) for each experimental group/condition, given as a discrete number and unit of measurement
☒	☐	A statement on whether measurements were taken from distinct samples or whether the same sample was measured repeatedly
☐	☒	The statistical test(s) used AND whether they are one- or two-sided<br>*Only common tests should be described solely by name; describe more complex techniques in the Methods section.*
☐	☒	A description of all covariates tested
☐	☒	A description of any assumptions or corrections, such as tests of normality and adjustment for multiple comparisons
☐	☒	A full description of the statistical parameters including central tendency (e.g. means) or other basic estimates (e.g. regression coefficient) AND variation (e.g. standard deviation) or associated estimates of uncertainty (e.g. confidence intervals)
☐	☒	For null hypothesis testing, the test statistic (e.g. *F*, *t*, *r*) with confidence intervals, effect sizes, degrees of freedom and *P* value noted<br>*Give P values as exact values whenever suitable.*
☐	☒	For Bayesian analysis, information on the choice of priors and Markov chain Monte Carlo settings
☒	☐	For hierarchical and complex designs, identification of the appropriate level for tests and full reporting of outcomes
☒	☐	Estimates of effect sizes (e.g. Cohen's *d*, Pearson's *r*), indicating how they were calculated

Our web collection on statistics for biologists contains articles on many of the points above.

Software and code

Policy information about availability of computer code

Data collection

All code is deposited in the following GitHub repository https://github.com/sudmantlab/amylase_diversity_project and is archived in zenodo (https://zenodo.org/doi/10.5281/zenodo.10995434).

Data analysis

Code for haplotype deconvolution can be found in the following GitHub repository https://github.com/raveancic/graph_genotyper and is archived in zenodo https://zenodo.org/doi/10.5281/zenodo.10843493. All other code used in the paper can be found in the following GitHub repository https://github.com/sudmantlab/amylase_diversity_project and is archived in zenodo (https://zenodo.org/doi/10.5281/zenodo.10995434).

All software programs used in this project and their versions are listed below:

BWA (v0.7.17)
Verkko (v1.3.1)
PGR-TK (v0.4.0)
Python
minimap2 (v2.26)
vep (v.105.0)
PGGB (v0.5.4)
ODGI (v0.8.3)
Snakemake (v7.32.3)
GAFPACK (https://github.com/ekg/gafpack, commit ad31875)
GFAINJECT (https://github.com/ekg/gfainject, commit f5feb7b)

cosigt (https://github.com/davidebolo1993/cosigt, commit e247261)
wgsim (https://github.com/lh3/wgsim, commit a12da33)
NGSNGS (https://github.com/RAHenriksen/NGSNGS, commit 559d552)
bwa-mem2 (https://github.com/bwa-mem2/bwa-mem2, commit 7f3a4db)
bcftools (v1.9)
vcftools (v0.1.16)
R (v4.2.1, v4.2.2)
plink (v1.90b6.21)
samtools (v1.17)
kalign (v3.3.5)
iqtree (v2.2.2.3)v
ggtree (v3.6.2)
cafe (v5.0.0)
ggplot (v3.4.2)
Adobe Illustrator (v27.5)
Adobe Indesign (v19.3)
selscan (v.2.0.2)
norm (v.1.3.0)
lassip (v.1.2.0)
ApproxWF (https://bitbucket.org/wegmannlab/approxwf/src/master/, commit 85793eb)
bmws (v0.1.0)
SLiM (v3.7.1)

For manuscripts utilizing custom algorithms or software that are central to the research but not yet described in published literature, software must be made available to editors and reviewers. We strongly encourage code deposition in a community repository (e.g. GitHub). See the Nature Portfolio guidelines for submitting code & software for further information.

Data

Policy information about availability of data

All manuscripts must include a data availability statement. This statement should provide the following information, where applicable:

- Accession codes, unique identifiers, or web links for publicly available datasets
- A description of any restrictions on data availability
- For clinical datasets or third party data, please ensure that the statement adheres to our policy

All data used in this project are publically available and described in the Datasets section of the methods. Copy number genotypes, structural haplotypes, haplotype deconvolutions, and pangenome graphs can be found in Supplementary Tables and a GitHub repository (https://github.com/sudmantlab/amylase_diversity_project) that is archived in zenodo (https://zenodo.org/doi/10.5281/zenodo.10995434). The HPRC data can be obtained at https://humanpangenome.org/data/. The 1000 genome data and the Human Genome Diversity Panel data can be obtained at https://www.internationalgenome.org/data/. The Simons Genome Diversity Panel data can be obtained at https://www.simonsfoundation.org/simons-genome-diversity-project/. The joint 1000 genome and the Human Genome Diversity Panel variant call set can be obtained at https://gnomad.broadinstitute.org/downloads#v3-hgdp-1kg. The ancient data are available on the European Nucleotide Archive under accession PRJEB64656 and PRJEB50857. The raw GTEx expression data can be obtained at https://gtexportal.org/home/datasets. GTEx genetic data are available under restricted access at https://gtexportal.org/home/protectedDataAccess.

Research involving human participants, their data, or biological material

Policy information about studies with human participants or human data. See also policy information about sex, gender (identity/presentation), and sexual orientation and race, ethnicity and racism.

Reporting on sex and gender	n/a
Reporting on race, ethnicity, or other socially relevant groupings	n/a
Population characteristics	n/a
Recruitment	n/a
Ethics oversight	n/a

Note that full information on the approval of the study protocol must also be provided in the manuscript.

Field-specific reporting

Please select the one below that is the best fit for your research. If you are not sure, read the appropriate sections before making your selection.

☐ Life sciences ☐ Behavioural & social sciences ☒ Ecological, evolutionary & environmental sciences

For a reference copy of the document with all sections, see nature.com/documents/nr-reporting-summary-flat.pdf

Ecological, evolutionary & environmental sciences study design

All studies must disclose on these points even when the disclosure is negative.

Study description	The structure and evolutionary history of the human amylase locus are described alongside it's population genetics.
Research sample	Worldwide human genetic data.
Sampling strategy	N/A
Data collection	N/A
Timing and spatial scale	N/A
Data exclusions	no data were excluded
Reproducibility	N/A
Randomization	N/A
Blinding	N/A

Did the study involve field work? ☐ Yes ☒ No

Reporting for specific materials, systems and methods

We require information from authors about some types of materials, experimental systems and methods used in many studies. Here, indicate whether each material, system or method listed is relevant to your study. If you are not sure if a list item applies to your research, read the appropriate section before selecting a response.

Materials & experimental systems

n/a	Involved in the study	
☒	☐	Antibodies
☒	☐	Eukaryotic cell lines
☒	☐	Palaeontology and archaeology
☒	☐	Animals and other organisms
☒	☐	Clinical data
☒	☐	Dual use research of concern
☒	☐	Plants

Methods

n/a	Involved in the study	
☒	☐	ChIP-seq
☒	☐	Flow cytometry
☒	☐	MRI-based neuroimaging

Plants

Seed stocks	*Report on the source of all seed stocks or other plant material used. If applicable, state the seed stock centre and catalogue number. If plant specimens were collected from the field, describe the collection location, date and sampling procedures.*
Novel plant genotypes	*Describe the methods by which all novel plant genotypes were produced. This includes those generated by transgenic approaches, gene editing, chemical/radiation-based mutagenesis and hybridization. For transgenic lines, describe the transformation method, the number of independent lines analyzed and the generation upon which experiments were performed. For gene-edited lines, describe the editor used, the endogenous sequence targeted for editing, the targeting guide RNA sequence (if applicable) and how the editor was applied.*
Authentication	*Describe any authentication procedures for each seed stock used or novel genotype generated. Describe any experiments used to assess the effect of a mutation and, where applicable, how potential secondary effects (e.g. second site T-DNA insertions, mosiacism, off-target gene editing) were examined.*
